# Investigation of Electric Field Induced Mixing in Silicon Micro Ring Resonators

**DOI:** 10.1038/s41598-017-03485-3

**Published:** 2017-06-13

**Authors:** Francesco De Leonardis, Richard A. Soref, Vittorio M. N. Passaro

**Affiliations:** 1 Photonics Research Group, Dipartimento di Ingegneria Elettrica e dell’Informazione, Politecnico di Bari Via Edoardo Orabona n. 4, 70125 Bari, Italy; 2 Department of Engineering, The University of Massachusetts, Boston, Massachusetts 02125 USA

## Abstract

In this paper we present a detailed theoretical investigation of the electric field induced mixing effect, in which the up and down frequency-conversion processes are obtained by inducing an effective second order susceptibility via the periodic spatial distribution of reversed biased p-i-n junctions. The possibility of realizing a frequency generation process within an integrated microring resonator is demonstrated here, by simulations, in the silicon on insulator platform. Furthermore, general physical features have been investigated by means of a comparative analysis of the frequency generation performance as a function of the input pump power, the linear and nonlinear losses, and the coupling factors. A conversion efficiency of 627.5 %/W has been obtained for the second harmonic generation process. Therefore, an improvement of 4 to 50 times with respect to the straight waveguides is achieved, depending on the cavity ring radius. Finally, for the up/down conversion, from telecom idler to mid-IR and from Mid-IR to telecom signal, respectively, an efficiency of 85.9%/W and 454.4 %/W has been obtained in the silicon microring resonator, respectively.

## Introduction

One of the main areas of current research in Group-IV technology is based upon nonlinear effects in photonic and optoelectronic circuits. In this sense, the demand for optical frequency generation is becoming increasingly evident. For manufacturing opto-electronic integrated circuits (OEICs) and photonic integrated circuits (PICs) upon silicon-on-insulator (SOI) substrates, the monolithic technological approach should be preferred over hybrid solutions. Indeed, total monolithic integration of group IV materials is expected, over the long term, to lower the costs of PICs and OEICs and to enhance their reliability with respect to those based on hybrid III-V-on-silicon platforms^1^. In the monolithic context, Four-Wave-Mixing (FWM) and Third-Harmonic-Generation (THG) amplification and optical processing have been demonstrated in recent years^2–10^, based on the third-order nonlinearities of silicon (Si) and germanium (Ge). However, it is expected that generating the lowest-order (second order) nonlinearity within those materials would enable a new array of CMOS-compatible optical devices capable of nonlinear functionalities such as electro-optic modulation, sum frequency up-conversion, and difference frequency generation. Therefore, it would be desirable to create ${\chi }^{(2)}$ in silicon-based Group-IV photonic circuits. Further, although centrosymmetric crystals such as Si and Ge lack second-order optical nonlinearity, several works have demonstrated that ${\chi }^{(2)}$ could be induced by breaking the symmetry via induced mechanical stress and strain^11–13^.

The Pockels effect has been experimentally and theoretically demonstrated in strained silicon^14–16^ as a promising candidate for realizing optical modulators and switches. In addition, there are proposals in the literature to investigate wavelength generation via ${\chi }^{(2)}$ in Si and Ge waveguides.

Second-Harmonic-Generation (SHG) experiments and first-principle calculations have been carried out at optical wavelengths in a Si waveguide by using a stressing silicon nitride overlayer^17, 18^. Furthermore, as Ge is largely recognized as a very promising material for extending the operation of PICs from the conventional near-infrared (NIR) wavelength range to the versatile mid-infrared (mid-IR) spectral window^19, 20^, we have recently investigated the possibility of inducing the SHG in strained germanium-on-silicon (GOS) waveguides, where the Ge centro-symmetry is broken by means of the asymmetric stress effect produced by a deposited SiN_x_ cladding film^21^. Moreover, a completely different approach has been proposed in literature in to induce SHG process^22^. In particular, the Si crystalline symmetry is broken when a direct current (DC) electric field is applied, inducing the ${\chi }^{(2)}$ susceptibility in a Si waveguide that is proportional to the large ${\chi }^{(3)}$ of Si. In addition, quasi-phase matching between the pump and second harmonic modes is achieved by controlling the spatial distribution of the DC field, and thus of ${\chi }^{(2)}$, via periodic distribution of reverse-biased p-i-n junctions. Thereby, a maximum efficiency of 12%/W at the pump wavelength of 2.29 µm has been recorded in a 1 mm long waveguide.

In this paper, we propose a generalization of the approach proposed in ref. 22. In particular, we theoretically investigate the Electric Field Induced Mixing (EFIM) effect in SOI waveguides, which is three-wave mixing (TWM) wherein the sum and difference frequency generation is obtained via an electric-field-induced ${\chi }^{(2)}$. In particular, our aim is to demonstrate the feasibility of an integrated microring resonator in order to perform spectral shifting (the TWM up/down conversion) with high conversion efficiency.

The paper is organized as follows. A detailed description of the mathematical modelling for the EFIM effect in an optical cavity is reported in Section 2. The validation of the proposed model is reported in Section 3.1, where a comparison with experimental results is performed in the case of the second harmonic generation process induced in straight waveguides. The theoretical calculations for the second harmonic generation and frequency conversion processes in SOI microring resonators are reported in Section 3.2 and 3.3, respectively. In particular, theoretical investigations of the conversion efficiency are presented as a function of coupling factors, linear and nonlinear losses, and input power. Finally, Section 4 summarizes the conclusions.

### Theoretical Background

**Figure 1:**
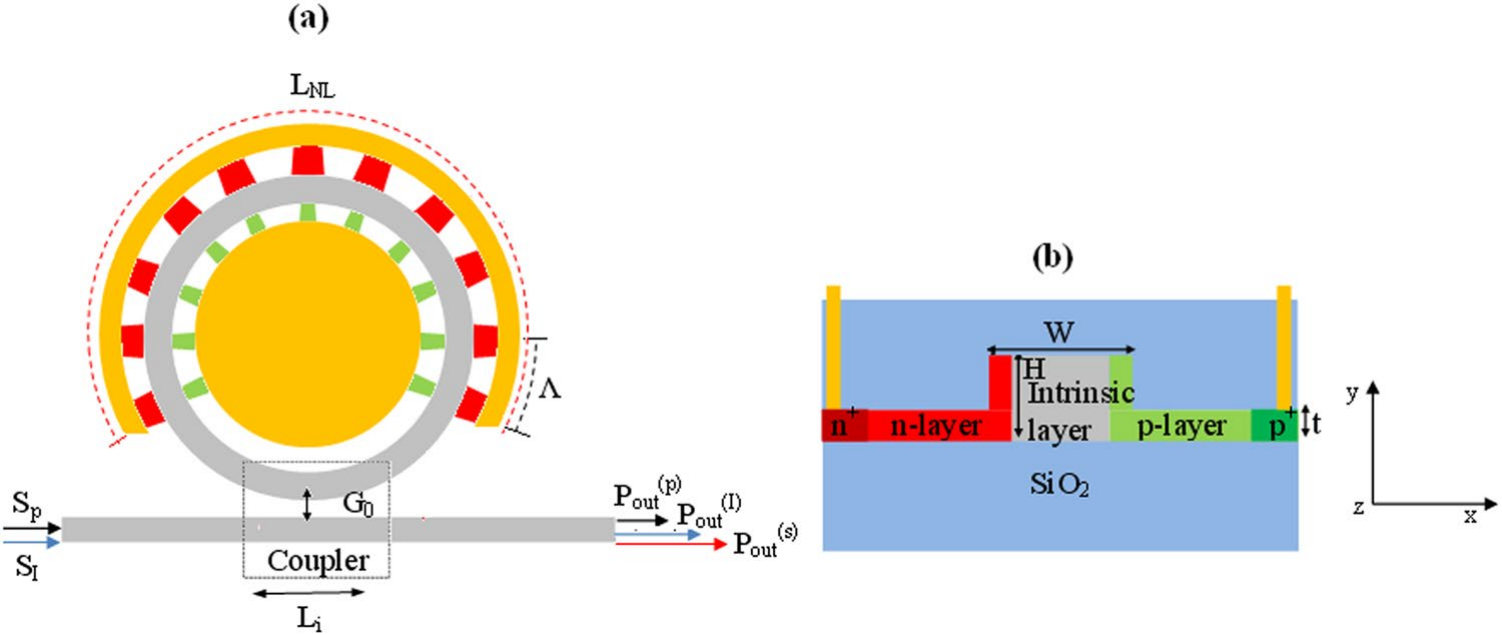
(**a**) Schematic of an integrated microring resonator inducing the EFIM process. The dashed box evidences the evanescent coupler (EC). The dimensions are not to scale. (**b**) Waveguide cross-section based on SOI technology platform. The high DC E_x_-field is present in the intrinsic waveguide core.

The ${\chi }^{(2)}$ susceptibility, which is proportional to ${\chi }^{(3)}$, can be induced in Si material by applying a DC electric field across the optical waveguide. In this section, we propose a theoretical model to investigate the electric field-induced mixing effect. The EFIM process could be interpreted as a particular case of FWM where one of the four electric fields involved in the process is a DC field used to induce the ${\chi }^{(2)}$ susceptibility inside the centro-symmetric semiconductor (the diamond cubic materials Si, Ge, 3C-SiC and C). As a result, the electric field-induced ${\chi }^{(2)}$ susceptibility is used to realize the TWM second order parametric process in which the pump and the idler optical waves are mixed in order to induce the sum frequency signal generation (SFG), i.e., ${\omega }_{1}+{\omega }_{3}={\omega }_{2}$, or the difference frequency generation (DFG), i.e., ${\omega }_{2}-{\omega }_{1}={\omega }_{3}$. In the analysis proposed in this paper, the potential of the EFIM effect induced in microring resonators (MRR) is investigated. With reference to Fig. 1(a), the input pump and idler waves, ${P}_{in}^{(p)}=\,{|{S}_{p}|}^{2}$ and ${P}_{in}^{(I)}=\,{|{S}_{I}|}^{2}$ which propagate into the input bus waveguide, are then coupled to the microring resonator by means of an evanescent-wave side coupler (EC) characterized by a gap, *G*_0_, and an interaction length, ${L}_{i}$. Therefore, they interact with the MRR nonlinear section obtained by realizing, over a length L_NL_, a periodic distribution of reversed biased p-i-n junctions, with a period *Λ* satisfying the quasi phase matching (QPM) condition for the EFIM process. Thus, the generation of the signal wave is obtained as a result of the optimized parametric process and the cavity enhancement effect.

In particular, the SOI technology platform is investigated here for the EFIM process. As an example, fully-etched waveguide cross-sections with a width *W* and height *H*, are considered and sketched in Fig. 1(b), where an intrinsic silicon layer is shown. Moreover, the side-region doped layers with a thickness, *t*, based on the Si semiconductor, are used to induce the ${\chi }^{(2)}$susceptibility via a reverse biased p-i-n junction. In this context, the equations that describe the power transfer among the pump (*p*), idler (*I*), and the signal (*s*) waves can be written as in Eqs (1–3), where ${a}_{1}$, ${a}_{2}$, and ${a}_{3}$ represent the slowly varying field amplitudes inside the circular optical cavity for the optical beams involved in the EFIM process.

1 $$\begin{array}{c}\frac{\partial {a}_{1}}{\partial t}=j{\rm{\Delta }}{\omega }_{1}{a}_{1}-\frac{1}{2}(\frac{1}{{\tau }_{c,1}}+\frac{1}{{\tau }_{l,1}}){a}_{1}-\frac{1}{2}{v}_{g,1}{\alpha }_{1}^{FCA}{a}_{1}-\frac{1}{2}{v}_{g,1}\frac{{\beta }_{1,1}^{TPA}}{{A}_{1,1}^{TPA}}{|{a}_{1}|}^{2}{a}_{1}\\ \quad \quad -\,{v}_{g,1}\frac{{\beta }_{1,2}^{TPA}}{{A}_{1,2}^{TPA}}{|{a}_{2}|}^{2}{a}_{1}-{v}_{g,1}\frac{{\beta }_{1,3}^{TPA}}{{A}_{1,3}^{TPA}}{|{a}_{3}|}^{2}{a}_{1}+j\frac{2\pi }{{\lambda }_{1}}{v}_{g,1}\Delta {n}_{1}{a}_{1}\\ \quad \quad +\,j{v}_{g,1}(\frac{{\gamma }_{1}}{{A}_{1,1}^{Kerr}}{|{a}_{1}|}^{2}+2\frac{{\gamma }_{1}}{{A}_{1,2}^{Kerr}}{|{a}_{2}|}^{2}+2\frac{{\gamma }_{1}}{{A}_{1,3}^{Kerr}}{|{a}_{3}|}^{2}){a}_{1}\\ \quad \quad +\,j{v}_{g,1}\,{f}_{1,2,3}\frac{{L}_{NL}}{\pi {L}_{cav}}\frac{{\omega }_{1}{\varepsilon }_{0}{c}_{0}^{3/2}{\mu }_{0}^{3/2}}{\sqrt{2}\sqrt{{n}_{eff,1}\cdot {n}_{eff,2}\cdot {n}_{eff,3}}}{\chi }_{eff}^{(2)}{a}_{2}{a}_{3}^{\ast }+j{\xi }_{1}{S}_{1}\end{array}$$

2 $$\begin{array}{c}\frac{\partial {a}_{2}}{\partial t}=j{\rm{\Delta }}{\omega }_{2}{a}_{2}-\frac{1}{2}(\frac{1}{{\tau }_{c,2}}+\frac{1}{{\tau }_{l,2}}){a}_{2}-\frac{1}{2}{v}_{g,2}{\alpha }_{2}^{FCA}{a}_{2}-\frac{1}{2}{v}_{g,2}\frac{{\beta }_{2,2}^{TPA}}{{A}_{2,2}^{TPA}}{|{a}_{2}|}^{2}{a}_{2}\\ \quad \quad -\,{v}_{g,2}\frac{{\beta }_{1,2}^{TPA}}{{A}_{1,2}^{TPA}}{|{a}_{1}|}^{2}{a}_{2}-{v}_{g,2}\frac{{\beta }_{2,3}^{TPA}}{{A}_{2,3}^{TPA}}{|{a}_{3}|}^{2}{a}_{2}+j\frac{2\pi }{{\lambda }_{2}}{v}_{g,2}{\rm{\Delta }}{n}_{2}{a}_{2}\\ \quad \quad +\,j{v}_{g,2}(\frac{{\gamma }_{2}}{{A}_{2,2}^{Kerr}}{|{a}_{2}|}^{2}+2\frac{{\gamma }_{2}}{{A}_{1,2}^{Kerr}}{|{a}_{1}|}^{2}+2\frac{{\gamma }_{2}}{{A}_{2,3}^{Kerr}}{|{a}_{3}|}^{2}){a}_{2}+j{v}_{g,2}\,{f}_{2,3,1}\frac{{L}_{NL}}{\pi {L}_{cav}}\\ \quad \quad \times \,\frac{{\omega }_{2}{\varepsilon }_{0}{c}_{0}^{3/2}{\mu }_{0}^{3/2}}{\sqrt{2}\sqrt{{n}_{eff,1}\cdot {n}_{eff,2}\cdot {n}_{eff,3}}}{\chi }_{eff}^{(2)}{a}_{1}{a}_{3}+j{\xi }_{2}{S}_{2}\end{array}$$

3 $$\begin{array}{rcl}\frac{\partial {a}_{3}}{\partial t} & = & j{\rm{\Delta }}{\omega }_{3}{a}_{3}-\frac{1}{2}(\frac{1}{{\tau }_{c,3}}+\frac{1}{{\tau }_{l,3}}){a}_{3}-\frac{1}{2}{v}_{g,3}{\alpha }_{3}^{FCA}{a}_{3}-\frac{1}{2}{v}_{g,3}\frac{{\beta }_{3,3}^{TPA}}{{A}_{3,3}^{TPA}}{|{a}_{3}|}^{2}{a}_{3}\\  &  & -\,{v}_{g,3}\frac{{\beta }_{1,3}^{TPA}}{{A}_{1,3}^{TPA}}{|{a}_{1}|}^{2}{a}_{3}-{v}_{g,2}\frac{{\beta }_{2,3}^{TPA}}{{A}_{2,3}^{TPA}}{|{a}_{2}|}^{2}{a}_{3}+j\frac{2\pi }{{\lambda }_{3}}{v}_{g,3}{\rm{\Delta }}{n}_{3}{a}_{3}\\  &  & +\,j{v}_{g,3}(\frac{{\gamma }_{3}}{{A}_{3,3}^{Kerr}}{|{a}_{3}|}^{2}+2\frac{{\gamma }_{3}}{{A}_{1,3}^{Kerr}}{|{a}_{1}|}^{2}+2\frac{{\gamma }_{3}}{{A}_{2,3}^{Kerr}}{|{a}_{2}|}^{2}){a}_{3}+j{v}_{g,2}\,{f}_{3,1,2}\frac{{L}_{NL}}{\pi {L}_{cav}}\\  &  & \times \,\frac{{\omega }_{3}{\varepsilon }_{0}{c}_{0}^{3/2}{\mu }_{0}^{3/2}}{\sqrt{2}\sqrt{{n}_{eff,1}\cdot {n}_{eff,2}\cdot {n}_{eff,3}}}{\chi }_{eff}^{(2)}{a}_{2}{a}_{1}^{\ast }+j{\xi }_{3}{S}_{3}\end{array}$$

The coefficients ${\beta }_{i,j}^{TPA}$ are the TPA coefficients, and ${\alpha }_{i}^{FCA}$ and ${\rm{\Delta }}{n}_{i}$ represent the free-carrier absorption (FCA) and the plasma dispersion effect as induced by the TPA process. Moreover, the coefficients ${\gamma }_{i}={n}_{2}({\omega }_{i})\cdot {\omega }_{i}/c$ take into account the self-phase modulation (SPM) and cross-phase modulation (XPM) effects as induced by the Kerr nonlinearity, where ${\omega }_{i}$ is the angular frequency of the *i* ^*th*^ beam inside the waveguide structure and ${n}_{2}$ is the nonlinear Kerr refractive index, which is evaluated as in ref. 23.

It is worth noting that the system of Eqs (1–3) holds for both DFG and SFG processes if frequencies ${\omega }_{1},\,{\omega }_{2}$, and ${\omega }_{3}$ are opportunely defined. In particular, taking ${\omega }_{p},\,{\omega }_{I}$, and ${\omega }_{s}$ as the angular pulsations of the pump, the telecom idler and the mid-IR signal, respectively, the EFIM DFG process is obtained by considering $1\to p$, $2\to I$, and $3\to s.$ Conversely, the $1\to p$, $2\to s$, and $3\to I$ condition is required in the EFIM SFG effect.

In Eqs (1–3), the effective nonlinear modal areas (${A}_{i,j}^{Kerr}={A}_{i,j}^{TPA}$) for the third-order nonlinearities are calculated according to the full-vectorial coupled mode theory (CMT)^10^. The term ${n}_{eff,i}$ is the effective refractive index at the wavelength of the *i* ^*th*^ beam inside the waveguide. Moreover, the coefficient ${f}_{i,j,k}$ ($i,j,k=1,2,3$) represents the overlap integral among pump, idler, and signal optical electric fields defined as in refs 24 and 25. The terms ${v}_{g,i}$ and ${\rm{\Delta }}{\omega }_{i}$ ($i=1,2,3$) indicate the group velocity and the mismatch from the resonance condition of the optical waves involved in the nonlinear process. Moreover, the coefficients ${\tau }_{c,i}$, and ${\tau }_{l,i}$ ($i=1,2,3$) represent the photon decay time inside the cavity, related to the ring-bus coupling process and intrinsic losses, respectively^26^. Furthermore, the coefficients ${\xi }_{i}$ are related to the power fraction transferred into the ring resonator from the input wave (${S}_{i}$)^26^. However, the system of Eqs (1–3) must be coupled with Eq. (4), i.e., the rate equation that governs the time dynamics of the free carrier density (${N}_{c}$). In this way, both the FCA and plasma dispersion effects can be calculated consistently^26, 27^.

4 $$\begin{array}{rcl}\frac{d{N}_{c}}{dt} & = & -\,\frac{{N}_{c}}{{\tau }_{eff}}+\frac{1}{2}[\frac{{\beta }_{1,1}^{TPA}\cdot {|{a}_{1}|}^{4}}{{({A}_{1,1}^{TPA})}^{2}\hslash {\omega }_{1}}+\frac{{\beta }_{2,2}^{TPA}\cdot {|{a}_{2}|}^{4}}{{({A}_{2,2}^{TPA})}^{2}\hslash {\omega }_{2}}+\frac{{\beta }_{3,3}^{TPA}\cdot {|{a}_{3}|}^{4}}{{({A}_{3,3}^{TPA})}^{2}\hslash {\omega }_{3}}\\  &  & +\,\frac{2\cdot {\beta }_{1,2}^{TPA}\cdot {|{a}_{2}|}^{2}\cdot {|{a}_{1}|}^{2}}{({A}_{1,2}^{TPA}\cdot {A}_{1,1}^{TPA})\hslash {\omega }_{1}}+\frac{2\cdot {\beta }_{1,2}^{TPA}\cdot {|{a}_{2}|}^{2}\cdot {|{a}_{1}|}^{2}}{({A}_{1,2}^{TPA}\cdot {A}_{2,2}^{TPA})\hslash {\omega }_{2}}+\frac{2\cdot {\beta }_{1,3}^{TPA}\cdot {|{a}_{3}|}^{2}\cdot {|{a}_{1}|}^{2}}{({A}_{1,3}^{TPA}\cdot {A}_{1,1}^{TPA})\hslash {\omega }_{1}}\\  &  & +\,\frac{2\cdot {\beta }_{1,3}^{TPA}\cdot {|{a}_{3}|}^{2}\cdot {|{a}_{1}|}^{2}}{({A}_{1,3}^{TPA}\cdot {A}_{3,3}^{TPA})\hslash {\omega }_{3}}+\frac{2\cdot {\beta }_{2,3}^{TPA}\cdot {|{a}_{2}|}^{2}\cdot {|{a}_{3}|}^{2}}{({A}_{2,3}^{TPA}\cdot {A}_{2,2}^{TPA})\hslash {\omega }_{2}}+\frac{2\cdot {\beta }_{2,3}^{TPA}\cdot {|{a}_{2}|}^{2}\cdot {|{a}_{3}|}^{2}}{({A}_{2,3}^{TPA}\cdot {A}_{3,3}^{TPA})\hslash {\omega }_{3}}]\end{array}$$

in Eq. (4), ${\tau }_{eff}$ is the carrier recombination effective lifetime.

The term ${\chi }_{eff}^{(2)}$ indicates the effective second order susceptibility responsible for the SFG or DFG processes, and it can be estimated as in the Method section.

## Results

### Validation

To test the mathematical model and the physical assumptions, we have particularized the EFIM effect considering the case of the second harmonic generation, and have compared our numerical results with the experimental measurements presented in the literature.

In particular, we have considered the case proposed in ref. 22, where the electric field-induced second harmonic generation (EFISHG) is induced in a silicon ridge waveguide.

In order to compare our results with the measurements proposed in ref. 22, we have to apply Eqs (1–4) to the straight waveguide case. This is possible after imposing the following conditions: i) nulling all terms depending on the coupling factors; ii) converting the terms in $\partial a/\partial t\to {v}_{g}\partial a/\partial z$, where *z* is the propagation direction; iii) setting ${a}_{1}$ = ${a}_{3}$ = ${a}_{p}$ (pump field amplitude), and ${a}_{2}$ = ${a}_{SH}$ (second harmonic field amplitude); iv) setting the boundary conditions at *z* = 0 as ${|{a}_{1}(0)|}^{2}$ = ${|{a}_{3}(0)|}^{2}$ = $0.5{P}_{in}$, and ${|{a}_{SH}(0)|}^{2}$ = 0. At this stage, the silicon waveguide cross section has been chosen to have: *W* = 800 nm, *H* = 220 nm, and *t* = 100 nm, as detailed in ref. 22. A spatially periodic electric field along the waveguide has been adopted to induce the quasi-phase matching between the pump and the second harmonic (SH) wave, propagating as quasi-TE modes at ${\lambda }_{p}=$ 2.29 µm and ${\lambda }_{SH}=$ 1.145 µm, respectively. The period $\Lambda$ = 1.443 µm has been selected to match two times the coherence length for the first order quasi-phase matching condition (see the Method section).

**Figure 2:**
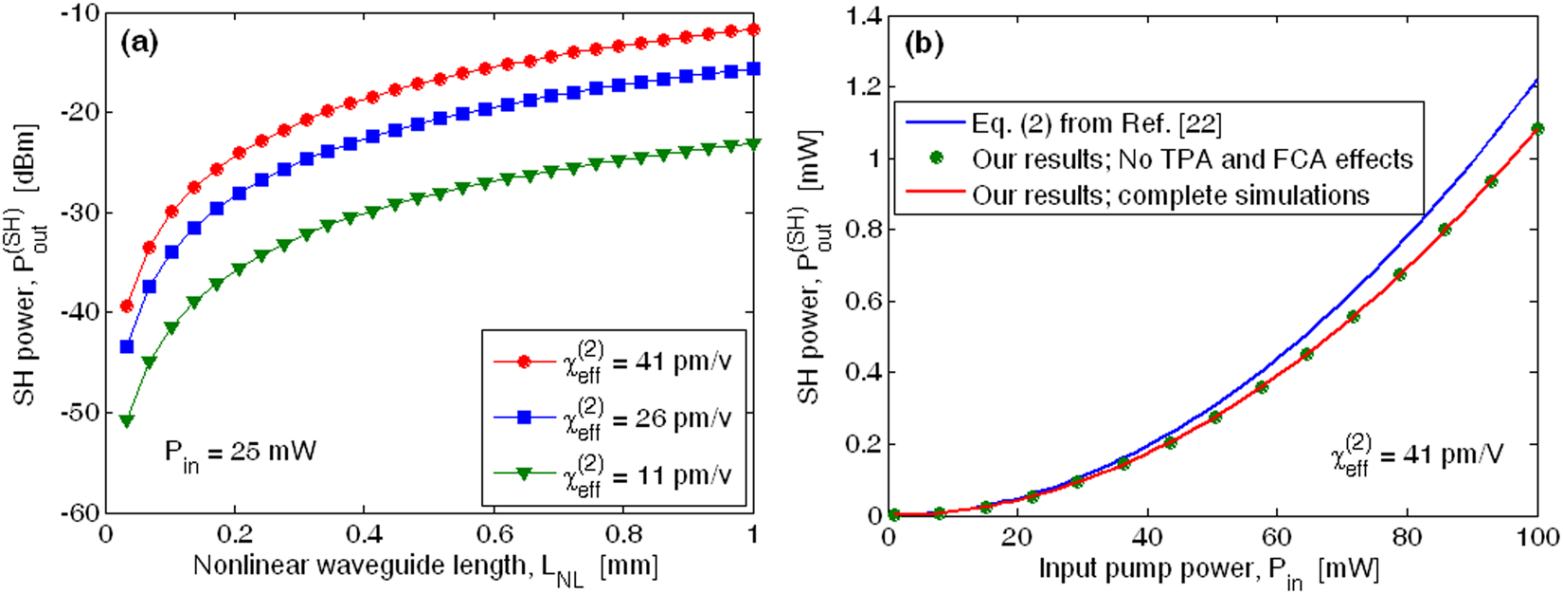
(**a**) SH power as a function of the nonlinear waveguide length for different values of the effective second order susceptibility. (**b**) SH power as a function of the input pump power.

Therefore, the analysis proposed in ref. 22 indicate that a maximum SHG conversion $\eta ={P}_{out}^{(SH)}/{P}_{in}^{2}$ of 13 ± 0.5 %/W has been obtained at ${\lambda }_{p}=$2.29 µm, that corresponds to an effective second order susceptibility ${\chi }_{eff}^{(2)}$ = 41 ± 1.5 pm/V. Figure 2(a) shows the SH power as a function of the nonlinear waveguide length ${L}_{NL}$ for different values of the effective second order susceptibility ${\chi }_{eff}^{(2)}$, assuming ${P}_{in}=$25 mW, and assuming pump and SH loss coefficients equal to ${\alpha }_{p}$ = 3.6 cm^−1^ and ${\alpha }_{SH}$ = 0.2 cm^−1^ ^22^. The agreement at ${L}_{NL}$ = 1 mm (as in the experimental setup of ref. 22) is demonstrated to be very good in the case of ${\chi }_{eff}^{(2)}$ close to 41 pm/V. A significant set of results is shown in Fig. 2(b), where the generated second harmonic power is plotted as a function of the input pump power in the case of ${\chi }_{eff}^{(2)}$ = 41 pm/V. In the plot, the curve obtained with Eq. (2) of ref. 22 is reported for comparison.

It is worth noting two fundamental aspects: First, due to the absence of the degenerate TPA effect at the pump wavelength^23^, the degenerate and nondegenerate TPA effects induced by the SH wave produce negligible influences on the second order parametric amplification, as can be seen by comparing the red curve with the circle markers, obtained by Eqs (1–4) when TPA and FCA are forced to zero. Second, for input pump power less than 20 mW, our numerical results are very close to those obtained by means of the formula of ref. 22 (Eq. (2)), assuming the undepleted regime. Conversely, the two theoretical approaches give previsions quite different when increasing the input pump power above 30 mW. Indeed, in the quasi-phase matching condition, the conversion efficiency can become appreciable at relatively high values of the input pump power. As a result, it is no longer possible to neglect the pump depletion as induced by the energy transfer to the SH wave. Thus, the accurate numerical solution of differential equations (1–4) is required. This comparison is also summarized in Table 1 where the measured and simulated output SH powers are reported.

**Table 1:** Comparison results for EFISHG effect in straight waveguide operating at pump and SH wavelength of 2.29 µm and 1.145 µm.

Method	SH output power [dBm]
Experimental results (Fig. 3 of ref. 22)	−11.86
Theoretical results (undepletion regime formula) (Eq. (2) of ref. 22)	−11.32
Theoretical results (our work)	−11.67

### EFISHG effect in microring resonator

Different from the inline architecture proposed in ref. 22 and simulated in the previous sub-section where the nonlinear device is represented by a simple SOI straight waveguide, in this section we theoretically analyze the possibility of inducing the EFISHG process in integrated microcavities based on SOI technology, where the microring resonator is evanescently coupled with an external bus waveguide. Our approach is motivated by the possibility that a microcavity—with its periodic spectral resonances–offers an increase in the parametric process efficiency due to resonant enhancement of the pump. In this sense, to clearly show the degree of improvement for the EFISHG efficiency, we assume, in our simulations, the same waveguide cross section and fundamental physical parameters as detailed in ref. 22.

It is worth noting that, to simulate the second harmonic generation process, we must opportunely modify Eqs (1–3). This is possible by imposing the following conditions: i) ${a}_{1}$ = ${a}_{3}$ = ${a}_{p}$ (pump field amplitude), and ${a}_{2}$ = ${a}_{SH}$ (second harmonic field amplitude); ii) ${S}_{p}$ = ${S}_{I}$ = $\sqrt{0.5{P}_{in}}$, and ${S}_{2}$ = 0 (see Fig. 1(a)). According to^22^, the SH signal at *λ*_*SH*_ = 1.145 µm can be obtained by assuming the pump power operating at *λ*_*p*_ =  2.29 µm and aligned as the TE_00_ mode. Although the pump does not suffer from any degenerate TPA effect (above the cut-off), the nonzero value of the degenerate and non-degenerate TPA coefficients, ${\beta }_{2,2}^{TPA}$ = 0.6 cm/GW, and ${\beta }_{1,2}^{TPA}$ = 0.5 cm/GW could induce detrimental effects on the generated SH wave. Indeed, it is reasonable to suppose that, if on the one hand the cavity enhancement effect improves the harmonic generation efficiency, on the other hand it could amplify the nonlinear absorption effect, thereby increasing both the pump depletion and the carrier accumulation inside the intrinsic waveguide. In this context, it is fundamental to simulate the p-i-n behaviour under the optical generation rate (*G*_*op*_). Numerical analysis of carrier recombination and carrier transport in a p-i-n junction has been carried out to obtain information about carrier density, DC electric field, screening electric field as induced by charge accumulation, and diode current density. Tibercad simulation software^28^ has been used for diode simulations. As an input of our simulations, we have defined the p-i-n diode geometry, doping regions and doping levels (Fig. 1(b)), as well as the reverse bias voltage. Thus, a parametric analysis changing the optical generation rate inside the waveguide cross section has been carried out, in order to investigate the influence of the TPA effect.

**Figure 3:**
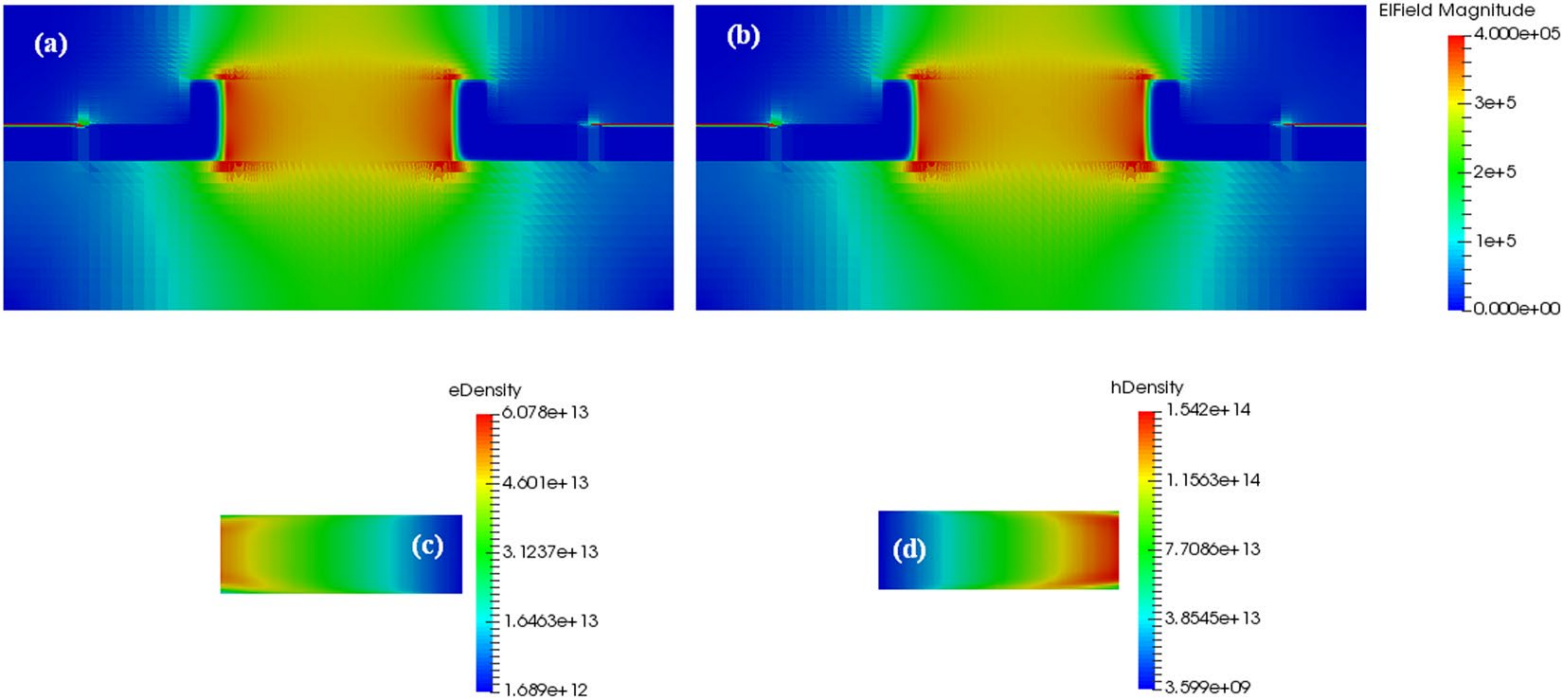
(**a**) DC electric field distribution, for *G*_*op*_ = 0 cm^−3^ s^−1^, and *V*_*bias*_ = −20 V. (**b**) DC electric field distribution, for *G*_*op*_ = 4 × 10^26^ cm^−3^ s^−1^, and *V*_*bias*_ = −20 V. (**c**) Zoom of the electron density distribution in the intrinsic Si layer, for *G*_*op*_ = 4 × 10^26^ cm^−3^ s^−1^, and *V*_*bias*_ = −20 V. (**d**) Zoom of the hole density distribution in the intrinsic Si layer, for *G*_*op*_ = 4 × 10^26^ cm^−3^ s^−1^, and *V*_*bias*_ = −20 V.

In this context, Fig. 3(a) and (b) show the DC electric field distribution inside the p-i-n diode, assuming *G*_*op*_ = 0 and 4 × 10^26^ cm^−3^ s^−1^, respectively.

**Figure 4:**
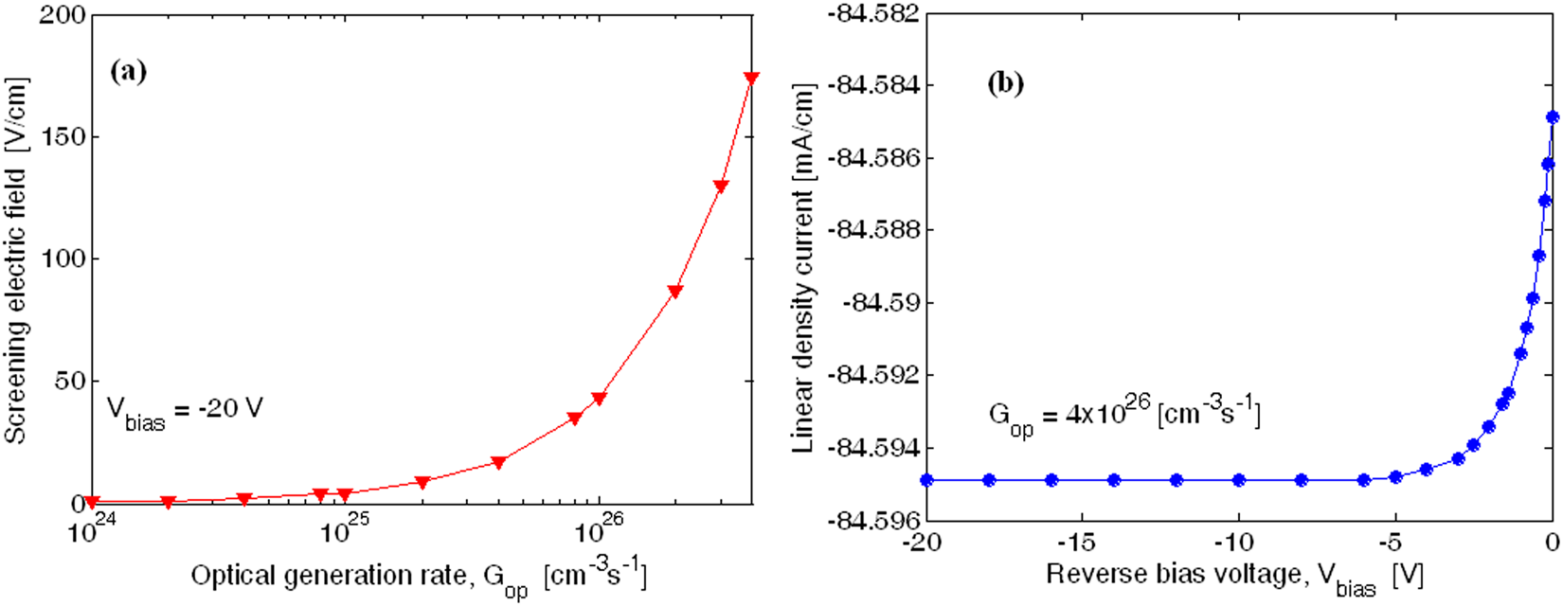
(**a**) Screening electric field as a function of the optical generation rate, for −20 V of bias voltage. (**b**) Linear density current at the p-i-n electrode as a function of the reverse bias voltage, for *G*_*op*_ = 4 × 10^26^ cm^−3^ s^−1^.

In addition, the doping level *N* = 1 × 10^18^ cm^−3^ for both n-layers and p-layers and ${V}_{bias}$ = −20 V have been considered in the simulations. Moreover, the electron and hole density distributions in the intrinsic layer are plotted in Fig. 3(c and d), for *G*_*op*_ = 4 × 10^26^ cm^−3^ s^−1^. The plots indicate that, although a very high optical generation rate *G*_*op*_ has been considered, the p-i-n diode works correctly since the carriers are swept away efficiently. As a result, the carrier accumulation inside the intrinsic waveguide region induces a low screening electric field, with negligible influence on ${\chi }_{eff}^{(2)}$. In detail, Fig. 4(a) shows the screening electric field as a function of the optical generation rate. Furthermore, the linear density current at the p-i-n electrodes as a function of the reverse bias voltage is plotted in Fig. 4(b), assuming again *G*_*op*_ = 4 × 10^26^ cm^−3^ s^−1^.

Although our simulations demonstrate that the presence of the reversed biased p-i-n diodes (intrinsic in the EFIM device) can positively contribute to a reduction of the effective recombination lifetime down to few picoseconds, hereafter we assume, as a postulate of the worst case, ${\tau }_{eff}$ = 0.25 ns, in order to take into account the influence of those ring sections without p-i-n junctions.

At this stage, to optimize the absolute power conversion efficiency ${\eta }_{ab}={P}_{out}^{(SH)}/{P}_{in}$, we first need to engineer the microcavity geometry so that the simultaneous dual resonant condition ${\rm{\Delta }}{\omega }_{p}=({\omega }_{p}-{\omega }_{p}^{0})=0$ and ${\rm{\Delta }}{\omega }_{SH}=({\omega }_{SH}-{\omega }_{SH}^{0})$ = 0 is achieved. A possible method is to design the cavity resonator to induce a distribution of the resonant angular frequencies, such as the difference ${\omega }_{SH}^{0}-{\omega }_{p}^{0}$ containing an integer number of Free Spectral Range values (FSRs). It is evident that this condition induces a discrete set of resonator ring radii $R$ to be used. In fact, developing the previous conditions, we obtain:

5 $$R=\frac{l\cdot c}{{\omega }_{p}^{0}(2{n}_{eff,SH}-{n}_{eff,p})}$$

where $l$ is the integer number indicating the difference between the longitudinal order for the pump and SH wave respectively. Thus, the number $l$ specifies the discrete set of cavity lengths to minimize the off-resonance effects. Rigorously speaking, the evaluation of ${\omega }_{p}^{0}$ in Eq. (5) requires the knowledge of $R$. Thus, to apply in a self-consistent way the previous equation, it is needed to implement a recursive procedure. For example, assuming *W* = 800 nm, *H* = 220 nm, *t* = 100 nm, *λ*_*p*_ = 2.29 µm and *λ*_*SH*_ = 1.145 µm, setting *l* = 2634 and applying the recursive procedure based on Eq. (5), we obtain a ring radius *R* = 249.97 μm. This value induces a mismatch with the ideal resonance condition as small as 6.13 and 1.13 pm for the pump and the SH wave, respectively. However, with this reduced mismatch the detrimental effects induced by the off-resonance operation condition can be considered negligible, and then the conditions ${\rm{\Delta }}{\omega }_{p}=0$ and ${\rm{\Delta }}{\omega }_{SH}$ = 0 can be adopted. The effects of resonance mismatch are discussed below.

Before proceeding with the theoretical investigations of the EFISHG processes, some comments on the loss coefficients are provided. With reference to the waveguide cross section sketched in Fig. 1(b), the linear loss coefficients include three different main contributions: ${\alpha }_{i}={\alpha }_{i}^{prop}+{\alpha }_{i}^{carrier}+{\alpha }_{i}^{scat}$, where the subscript *i* is relevant to the *i* ^*th*^ wave involved in the EFISHG process. Actually, ${\alpha }_{i}^{prop}$ is related to the propagation loss coefficient evaluated for the intrinsic Si waveguide. The coefficients ${\alpha }_{i}^{carrier}$ depend on the carrier distribution induced by the p-i-n diode. According to the procedure used in ref. 22, the carrier distribution within the silicon waveguide is calculated by means of diode simulations performed by Tibercad software^28^. The carrier distribution is converted into changes of the real and imaginary part of the silicon refractive index by the relationships given in ref. 27. Thus optical simulations performed by FEM method lead us to extract the loss coefficients ${\alpha }_{i}^{carrier}$ from the complex propagation constants. Our investigations indicate that the ${\alpha }_{i}^{carrier}$ coefficient strongly increases by decreasing the waveguide width *W*, and increasing the wavelength *λ*, recording values very close to those obtained in ref. 22. Thus consistently, we can set in the following: ${\alpha }_{p}^{carrier}$ ≈ 3.6 cm^−1^ (15.5 dB/cm), and ${\alpha }_{SH}^{carrier}$ ≈ 0.2 cm^−1^ (0.9 dB/cm). Moreover, light scattering at sidewall roughness is generally considered to be a significant loss source in ring resonator. Due to the nature of the lithography fabrication process, roughness on the vertical sidewalls of the waveguide is unavoidable. The losses are correlated with the periodicity of the roughness as well as its dimensions, and scale dramatically with the index contrast. Thus, a reduction of the scattering loss can be obtained by lowering the overlap of the optical mode with the vertical sidewalls. In this context, two different approaches can be considered: using SOI wire waveguide with smaller height and larger width; using a rib waveguide defined by a partial etch, which also has less sidewall surface, and weaker confinement. For example, the case of a wire waveguide with a height of only 100 nm and a width of 600 nm shows a reduced scattering loss^29^. Recent advances in process technology have brought the losses down to 2–3 dB/cm with air cladding and less than 2 dB/cm with oxide cladding^29^. It is worth outlining that the waveguide cross section used in this section presents all the necessary conditions (oxide cladding, partial etch, small rib height and larger width) to be confident that the scattering losses are not critical. Thus by following a conservative approach, we assume the scattering losses of about 2 dB/cm for both pump and SH waves, respectively. In this context, assuming ${\alpha }_{i}^{prop}$ to be 0.5 dB/cm, the average values for the linear loss coefficient can be estimated in ${\alpha }_{p}$ ≈ 4.1 cm^−1^ and ${\alpha }_{SH}$ ≈ 0.7 cm^−1^.

**Figure 5:**
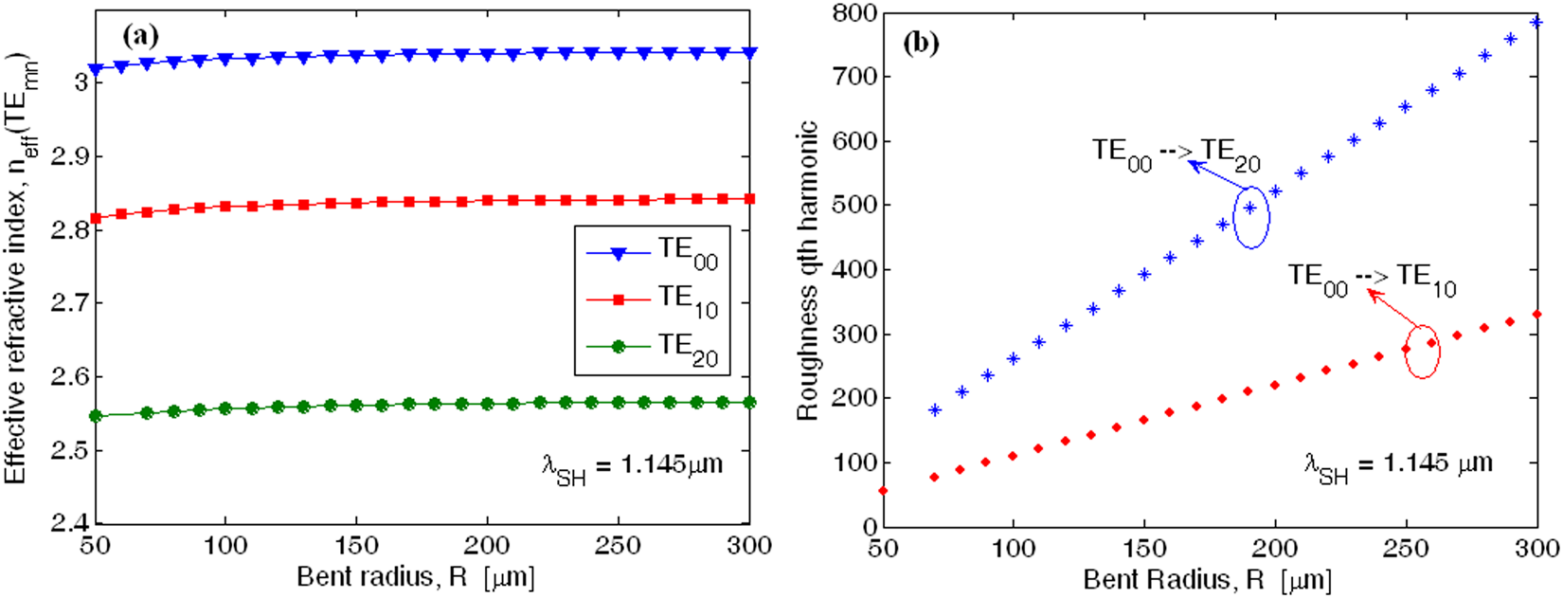
(**a**) Effective refractive index as a function of the bent radius for the fundamental and higher order quasi-TE modes at *λ*_*SH*_ = 1.145 µm; (**b**) Roughness *qth* harmonic ring radius combination inducing phase matching condition between fundamental and higher order quasi-TE modes.

Figure 5(a) shows the effective refractive index at *λ*_*SH*_ = 1.145 µm as a function of the bend radius $R$ for the fundamental (TE_00_) and higher order (TE_mn_) quasi-TE modes, respectively.

The plot indicates that the bending influence is negligible for ring radius larger than 80 µm. In particular, the difference between the effective refractive index for fundamental and higher order quasi-TE modes ($${\rm{\Delta }}{n}_{eff}^{(T{E}_{00}-T{E}_{mn})}={n}_{eff}^{(T{E}_{00})}-{n}_{eff}^{(T{E}_{mn})}$$) becomes independent from $$R$$. However, ring resonators based on multi-modal waveguides should suffer from the mode coupling as induced by lateral roughness. The sidewall imperfections induce a random change, $$\delta \varepsilon $$, in the permittivity profile with respect to the ideal smooth waveguide. From a mathematical point of view, this random function can be considered periodic with a fundamental period given by the ring circumference. Under this condition, $$\delta \varepsilon $$ can be decomposed into Fourier series expansion as a function of the curvilinear coordinate ($$s)$$ along the ring, $$\delta \varepsilon (s)=\sum _{q}\delta {\varepsilon }_{q}{e}^{\frac{jq\cdot 2\pi \cdot s}{2\pi R}}$$, where $$\delta {\varepsilon }_{q}$$ is the amplitude of the *qth* harmonic, $$q=0,\pm 1,\pm 2,\ldots $$ ^30, 31^. In this context, the *qth* harmonic produces a phase term given by $$q/R$$. which, in turn, can induce the phase matching condition between the fundamental and higher order quasi-TE modes, if the following relationship $${\rm{\Delta }}{n}_{eff}^{(T{E}_{00}-T{E}_{mn})}=\lambda q/(2\pi R)$$ is satisfied. As a result, the induced mode-coupling could increase the optical loss. In Fig. 5(b) we show the pair of values for *q*-order harmonic and ring radius satisfying the phase matching for the modal coupling $$T{E}_{00}\to T{E}_{10}$$ and $$T{E}_{00}\to T{E}_{20}$$, respectively. The plot evidences that for large ring radius ($$R > 200\mu m$$), as it will be used in our devices, the harmonic order involved in the phase matching is larger than 200 or 500, for $$T{E}_{00}\to T{E}_{10}$$ and $$T{E}_{00}\to T{E}_{20}$$, respectively. Moreover, considering that the strength of the harmonic amplitude reduces with increasing the *q-*value, we can guess that the modal-coupling-induced losses are negligible with respect to the other contributions before mentioned. It worth outlining that similar analysis is not reported at the pump wavelength because the waveguide satisfies the mono-modal condition.

**Figure 6:**
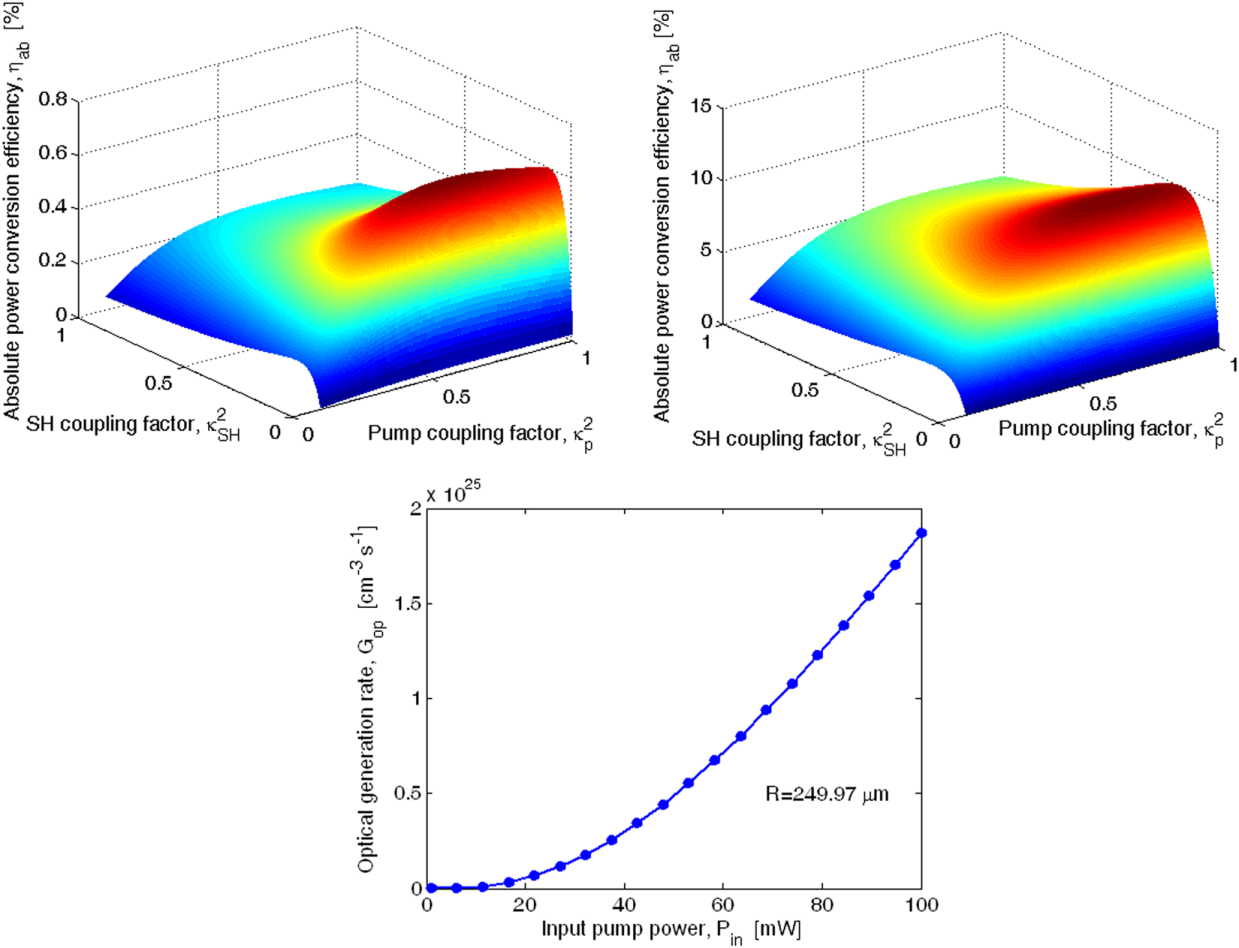
Absolute power conversion efficiency as a function of the pump and SH coupling factor: (**a**) *P*_*in*_ = 1 mW. (**b**) *P*_*in*_ = 25 mW. (**c**) TPA-induced optical rate generation as a function of the input pump power.

Following Eqs (1–4), a parametric investigation of the EFISHG efficiency in microring resonators as a function of the power coupling factors for the pump and second harmonic wave, indicated as ${\kappa }_{p}^{2}$ and ${\kappa }_{SH}^{2}$, respectively, has been performed. In particular, the absolute power conversion efficiency, ${\eta }_{ab}$, has been initially considered in simulations for two different input pump powers: ${P}_{in}\,$ = 1 mW for the undepleted regime, and ${P}_{in}\,$ = 25 mW for the depletion condition. The results are plotted in Fig. 6a,b), respectively. Furthermore, the quasi-TE state of polarization for both pump and SH waves has been considered in order to maximize the overlap integral, according to the p-i-n junction orientation (see Fig. 1). The 3D plots refer to a nonlinear device having ${\chi }_{eff}^{(2)}$ = 41 pm/V, ${L}_{NL}$ = 1 mm and $\Lambda$ = 1.4435 µm, as required by the QPM condition.

Thus, setting ${\alpha }_{p}$ = 4.1 cm^−1^ and ${\alpha }_{SH}$ = 0.7 cm^−1^ the unloaded cavity quality factor results in ${Q}_{p}^{l}$ = 7.54 × 10^3^ and ${Q}_{SH}^{l}$ = 1.2 × 10^5^, respectively.

As is known^26^, the coupling condition can be described in terms of the ratio $K=\,{\tau }_{l}/{\tau }_{c}$ between the intrinsic resonator and the coupling decay time^26^. Following the standard conventions, undercoupling is denoted by $K$<1, overcoupling by $K$>1, and critical coupling by $K$ = 1. This last condition corresponds, in the case of the MRR under investigation, to coupling factor values of ${\kappa }_{p,cr}^{2}$ = 0.644 and ${\kappa }_{SH,cr}^{2}$ = 0.1 for the pump and SH wave, respectively.

The investigation carried out in Fig. 6 leads to some important design considerations: i) an optimum combination between the pump and SH coupling factors, indicated as ${\kappa }_{p,op}^{2}$ and ${\kappa }_{SH,op}^{2}$, induces a peak for the SHG efficiency; ii) the maximum value of ${\eta }_{ab}$ (${\eta }_{ab}^{max}$) increases when moving from the undepleted regime towards the depletion condition. In particular, we record ${\eta }_{ab}^{max}$ = 0.65%, and ${\eta }_{ab}^{max}$ = 10.16%, for ${P}_{in}\,$ = 1 mW, and 25 mW, respectively; iii) the ${\eta }_{ab}^{max}$ value is obtained for values of ${\kappa }_{p}^{2}$ and ${\kappa }_{SH}^{2}$ satisfying the critical coupling or the overcoupling condition, depending on the undepleted or depleted regime, respectively. In particular, our simulations indicate that the maximum efficiency is obtained for ${\kappa }_{p,op}^{2}$ = 0.652, and ${\kappa }_{SH,op}^{2}$ = 0.11 (close to the critical coupling condition), for ${P}_{in}\,$ = 1 mW, and ${\kappa }_{p,op}^{2}$ = 0.76 and ${\kappa }_{SH,op}^{2}$ = 0.18 for ${P}_{in}\,$ = 25 mW. As a result, in the depletion regime the critical coupling condition for both pump and SH waves is not the best way to excite the SHG parametric process. In Fig. 6(c) the optical generation rate induced by the TPA coefficients ${\beta }_{2,2}^{TPA}$, and ${\beta }_{1,2}^{TPA}$ is plotted as a function of the input pump power. The plot indicates that the calculated ${G}_{op}$ is smaller than the values of 4 × 10^26^ cm^−3^ s^−1^, assumed in the p-i-n diode simulations (see Fig. 3), confirming that the MRR EFISHG device operating with ${P}_{in}\,$ = 25 mW does not suffer from any detrimental effects induced by the screening electric field.

In any case, it is crucial to note that, unlike the undepleted condition in which the critical coupling factors can be easily estimated from both the intrinsic losses and the resonator length, in the depletion regime (high input pump power) it is erroneous to estimate the peak position from the relationship ${\tau }_{l}={\tau }_{c}$. Thus, numerical solution of the equations (1–4), is needed in order to determine the values ${\kappa }_{p,op}^{2}$ and ${\kappa }_{SH,op}^{2}$ for a given input pump power. However, it is not easy to simultaneously obtain ${\kappa }_{p,op}^{2}$, and ${\kappa }_{SH,op}^{2}$ by considering a single evanescent coupler. Indeed with reference to the architecture sketched in Fig. 1, we have only the coupling gap between the MRR and the bus waveguide to be engineered to realize the dual condition, i.e. ${\kappa }_{p}^{2}$ = ${\kappa }_{p,op}^{2}$ and ${\kappa }_{SH}^{2}$ = ${\kappa }_{SH,op}^{2}$. As a result, some deviations from the optimal design must be taken into account.

**Figure 7:**
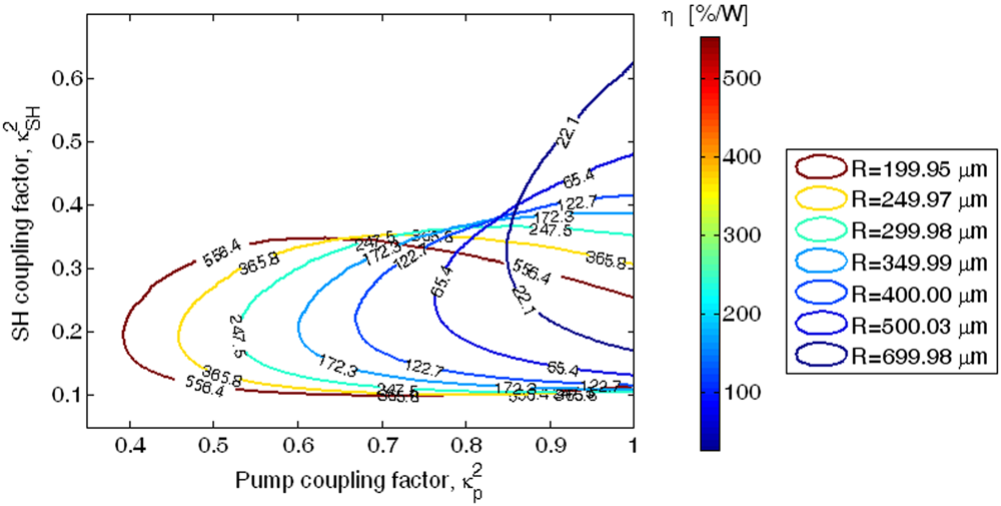
Contour curves at 90% of maximum value of the efficiency *η* as a function of the pump and SH coupling factors, for different values of MRR radius.

In this sense, additional investigations are also proposed in Fig. 7, where the contour curves at 90% of the maximum value (${\eta }^{max}$) of the efficiency $\eta$ ($\eta ={P}_{out}^{(SH)}/{P}_{in}^{2}$), are plotted as a function of ${\kappa }_{p}^{2}$ and ${\kappa }_{SH}^{2}$, for different values of the ring radius ranging from 200 to 700 µm, and satisfying the dual resonant condition. In the simulations, an input pump power ${P}_{in}\,$ = 25 mW has been assumed.

As a result, the plot indicates that for a fixed value of the pair (${\kappa }_{SH}^{2}$, ${\kappa }_{p}^{2}$) located inside the contour curves, an efficiency $\eta$ larger than ${\eta }^{90 \% }=0.9\times \,{\eta }^{max}$ can be always achieved. Moreover, Fig. 7 shows that the value of ${\eta }^{90 \% }$decreases from 556.4 %/W to 22.1 %/W when increasing the ring radius from 199.95 to 699.98 µm, respectively, as a result of the reduction of the enhancement cavity factor ($\Gamma ={|{a}_{p}|}^{2}/{P}_{in}$). In addition, the level curves move towards larger values of ${\kappa }_{p}^{2}$ and ${\kappa }_{SH}^{2}$, enclosing a smaller area as the cavity length is increased.

**Figure 8:**
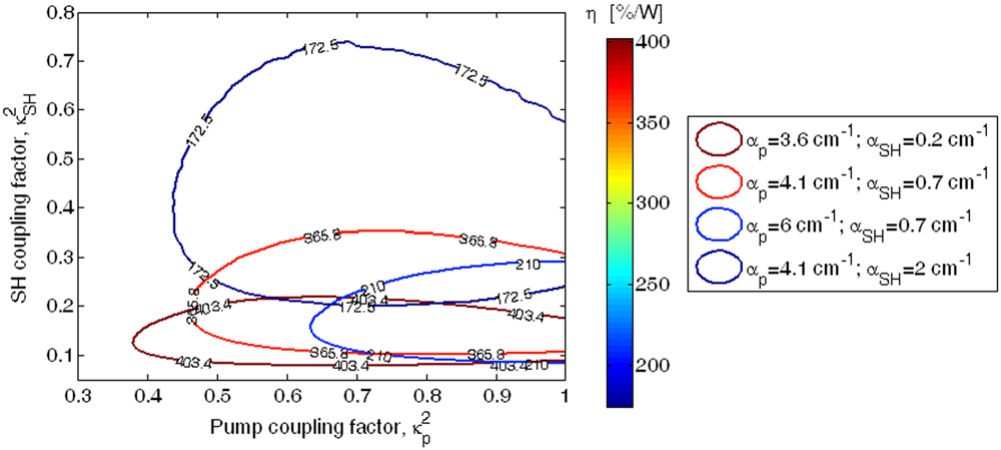
Contour curves at 90% of maximum value of the efficiency *η* as a function of the pump and SH coupling factor, for different values of pump and SH loss coefficient.

A similar investigation is also proposed in Fig. 8 where the level curves ${\eta }^{90 \% }$ are given as a function of ${\kappa }_{p}^{2}$ and ${\kappa }_{SH}^{2}$ for different values of the pump and SH loss coefficients, assuming $R$ = 249.97 µm, and ${P}_{in}\,$ = 25 mW. The plot indicates that for a fixed value of ${\alpha }_{SH}$ and decreasing the ${\alpha }_{p}$ value, the level curves shift towards the region of the plane (${\kappa }_{p}^{2}$, ${\kappa }_{SH}^{2}$) characterized by lower and larger values of ${\kappa }_{p}^{2}$ and ${\kappa }_{SH}^{2}$, respectively. In addition, an increasing of the enclosed area can be observed. The opposite trend is recorded for a fixed value of ${\alpha }_{p}$ and decreasing the ${\alpha }_{SH}$ value. Thus, our simulations indicate that ${\eta }^{90 \% }$ ranges from 403.4 %/W to 172.5 %/W, for ${\alpha }_{p}$ = 3.6 cm^−1^, ${\alpha }_{SH}$ = 0.2 cm^−1^ and ${\alpha }_{p}$ = 4.1 cm^−1^, ${\alpha }_{SH}$ = 2 cm^−1^.

The analysis of the efficiency ${\eta }^{90 \% }$ as a function of pump and SH power coupling factors allows us to find important design guidelines for guided-wave evanescent couplers (ECs). Indeed, any shape and architecture of the ECs can be practically designed and fabricated with the only requirement being that the pair (${\kappa }_{SH}^{2}$, ${\kappa }_{p}^{2}$) must be chosen in order to achieve specific parametric process performance (e.g., $\eta \ge$ ${\eta }^{90 \% }$).

It is worth pointing out that the simulations performed to obtain Figs (6 and 7) are very time consuming due to the solution of the differential equation system for each value of ${\kappa }_{p}^{2}$ and ${\kappa }_{SH}^{2}$. In this context, the efficiency ${\eta }^{90 \% }$ as a function of pump and SH power coupling factors could be more quickly obtained if TPA, SPM, XPM, FCA, and plasma dispersion effects were forced to zero. Under these assumptions, Eqs (1–3) in the steady-state condition yield the set of Eqs (6 and 7):

6 $${P}_{out}^{(SH)}=\frac{{\kappa }_{SH}^{2}{({v}_{g,SH}\cdot {\gamma }_{SH})}^{2}{P}_{p}^{2}}{[{(0.5{\tau }_{SH}^{-1})}^{2}+{\rm{\Delta }}{\omega }_{SH}^{2}]}$$

in Eqs (6), ${P}_{p}$, and ${P}_{out}^{(SH)}$ are the intra-cavity pump power and the generated SH power at the output external bus, respectively. In addition, ${P}_{p}$ must satisfy Eq. (7):

7 $$\begin{array}{c}{P}_{p}^{3}\frac{{({v}_{g,SH}\cdot {\gamma }_{SH})}^{2}{({v}_{g,p}\cdot {\gamma }_{p})}^{2}}{[{(0.5{\tau }_{SH}^{-1})}^{2}+{\rm{\Delta }}{\omega }_{SH}^{2}]}+2{P}_{p}^{2}\frac{({v}_{g,SH}\cdot {\gamma }_{SH})({v}_{g,p}\cdot {\gamma }_{p})}{[{(0.5{\tau }_{SH}^{-1})}^{2}+{\rm{\Delta }}{\omega }_{SH}^{2}]}\\ \times \quad [(0.25{\tau }_{SH}^{-1}{\tau }_{p}^{-1})-{\rm{\Delta }}{\omega }_{SH}{\rm{\Delta }}{\omega }_{p}]+{P}_{p}[{(0.5{\tau }_{p}^{-1})}^{2}+{\rm{\Delta }}{\omega }_{p}^{2}]-\frac{{v}_{g,p}}{{L}_{cav}}{k}_{p}{S}_{p}\end{array}$$

where

8 $${\gamma }_{SH}={f}_{p,p,SH}\frac{{L}_{NL}}{\pi {L}_{cav}}\frac{{\omega }_{SH}{\varepsilon }_{0}{c}_{0}^{3/2}{\mu }_{0}^{3/2}}{\sqrt{2}\sqrt{{n}_{eff,p}\cdot {n}_{eff,p}\cdot {n}_{eff,SH}}}{\chi }_{eff}^{(2)}$$

At this step, it is worth outlining that Eqs (6 and 7) are strictly valid in the wavelength range in which all TPA effects are above the cut-off, and the Kerr effect manifests itself over a scale length larger than that of the second order parametric process. In other words, Eqs (6 and 7) can be used considering that they overestimate the efficiency but at the same time represent an efficient tool to have preliminary information about the optimal region in the plane (${\kappa }_{SH}^{2}$, ${\kappa }_{p}^{2}$).

The generalized model implemented here makes it possible to simulate and design any desired EC. According to our previous work^32^, the model used and presented here can be considered a very robust and general approach for the investigation and design of an integrated directional coupler based on photonic waveguides. This approach can be applied to a very large variety of bend paths characterizing the transition lengths (i.e., arc, S-bend, cosine, sine, and so on), as well as several photonic waveguide architectures (e.g., rib, slot, wire, and so on), without any limitation on coupler geometrical parameters and configuration, waveguide cross sections and technology platform. For example, an EC based upon a circular section and a straight waveguide (as in Fig. 1(a)) has been employed in this investigation, where the coupler gap dependence of the propagation direction has been calculated as a function of the circumference arc involved in the coupling process^32^. Thus, we select three kinds of EC configurations (named EC#1, EC#2, EC#3). In particular, EC#1, and EC#2 induce an efficiency $\eta$ close to contour curve ${\eta }^{90 \% }$ while, and EC#3 produces an $\eta$ value outside the contour curve, respectively. It is worth noting that assuming $R$ = 249.97 µm, values for ${G}_{0}$ do not exist to induce the dual condition, i.e. ${\kappa }_{p}^{2}$ = ${\kappa }_{p,op}^{2}$ and ${\kappa }_{SH}^{2}$ = ${\kappa }_{SH,op}^{2}$. The evanescent coupler parameters in terms of $R$, ${G}_{0}$, and interaction length ${L}_{i}$, are summarized in Table 2.

**Table 2:** Pump and SH coupling factors at 2.29 µm and 1.145 µm.

Evanescent coupler parameters	Coupling power factors	
	${{\boldsymbol{\kappa }}}_{{\boldsymbol{p}}}^{{\bf{2}}}$	${{\boldsymbol{\kappa }}}_{{\boldsymbol{SH}}}^{{\bf{2}}}$
EC#1: *R* ≅ 250 µm, ${G}_{0}$ = 52 nm,$\,{L}_{i}\cong$ 34 µm	0.775	0.1
EC#2: $R$ ≅ 250 µm, ${G}_{0}$ = 66 nm,$\,{L}_{i}$ $R$ ≅ 43 µm	0.59	0.080
EC#3: $R$ ≅ 250 µm, ${G}_{0}$ = 80 nm,$\,{L}_{i}$ $R$ ≅ 52 µm	0.4	0.0645

**Figure 9:**
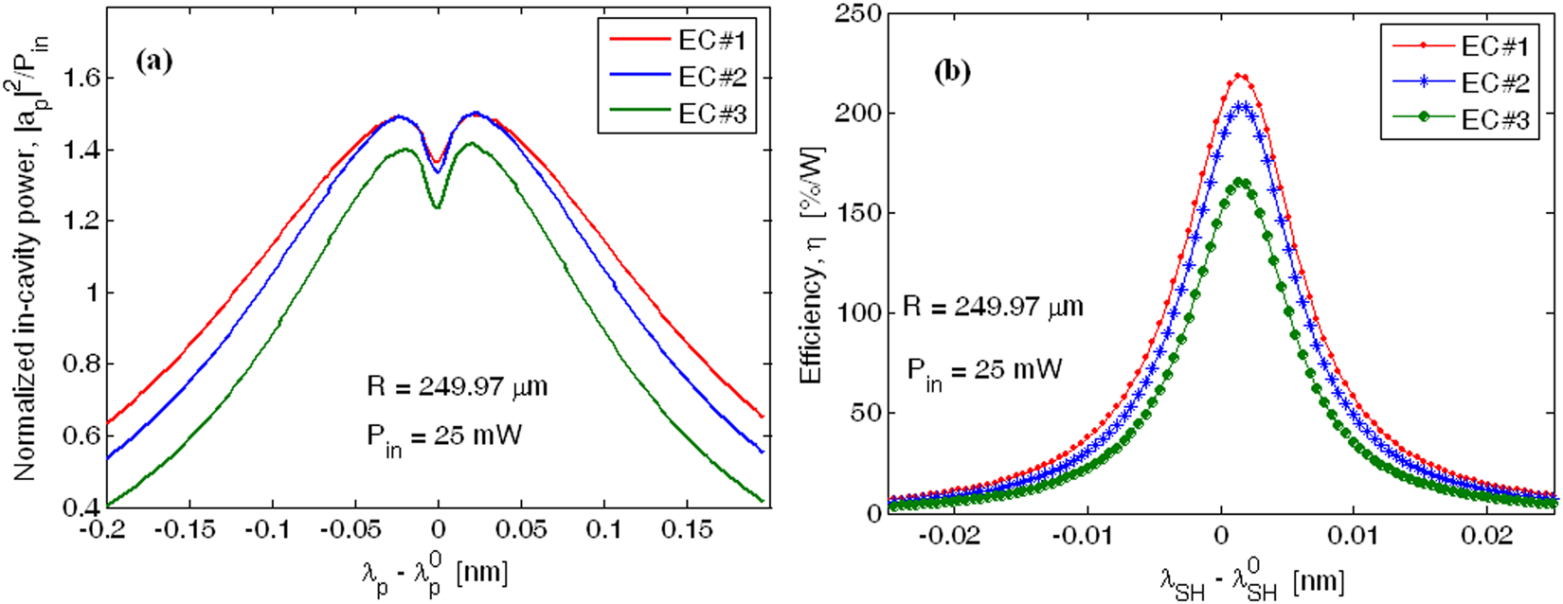
Spectrum as a function of the wavelength mismatch. (**a**) Normalized in-cavity pump power. (**b**) SH efficiency.

At this stage of the analysis, the influence of the mismatch between the input pump wavelength and the cavity resonances $({\lambda }_{p}-{\lambda }_{p}^{0})$ as well as the mismatch between the generated SH wavelength and the cavity resonances $({\lambda }_{SH}-{\lambda }_{SH}^{0})$, is worthy of investigation. To this purpose, the normalized in-cavity pump power $({|{a}_{p}|}^{2}/{P}_{in})$ as a function of the pump wavelength mismatch is plotted in Fig. 9(a), assuming the ECs as listed in Table 2. In addition, ${\alpha }_{p}$ = 4.1 cm^−1^, ${\alpha }_{SH}$ = 0.7 cm^−1^, $R$ = 249.97 µm, and ${P}_{in}\,$ = 25 mW have been assumed in our simulations. It is important that the period of the p-i-n distribution is set equal to $\Lambda$ = 1.4435 µm for realizing the quasi-phase matching condition for pump and SH quasi-TE modes at ${\lambda }_{p}^{0}$ and ${\lambda }_{SH}^{0}$, respectively. The plot reveals the presence of a dip close to ${\lambda }_{p}-{\lambda }_{p}^{0}=0$ as a result of the pump depletion due to the energy transfer to the SH wave. However, the dip depth is also strongly influenced by the coupling factors. In particular two opposite trends can be outlined: i) lower values of ${\kappa }_{SH}^{2}$ produce higher values of the in-cavity SH power (see Table 3), thereby inducing an increasing of the pump depletion inside the cavity resonator; ii) lower values of ${\kappa }_{SH}^{2}$ reduce the SH power extraction from the MRR towards the external bus, thus inducing a degradation in the nonlinear efficiency at the output waveguide. Therefore, the combination of these effects produces the curves of Fig. 9(a and b). For example comparing EC#1 and EC#3, we can observe that EC#3 presents a smaller SH efficiency $\eta$, although the in-cavity pump power shows a deeper dip, indicating a larger energy trasfer to the in-cavity SH wave. Moreover, Fig. 9(b) shows a spectrum red shift as a result of the opposite trend induced by SMP, and XPM processes (red shift) and the plasma dispersion effect (blue shift). The spectral characterisics for both pump and SH waves are summarized in Table 3.

**Table 3:** Spectral parameters for Pump and SH waves.

Evanescent coupler	Pump wave			
	FWHM [nm]	Overall quality factor *Q*	In-cavity dip power [mW]	Enhancement factor
EC#1	0.348	6.84 × 10^3^	3.29	1.5
EC#2	0.305	7.87 × 10^3^	4.14	1.51
EC#3	0.252	9.30 × 10^3^	4.43	1.41
**Evanescent coupler**	**Second Harmonic wave**			
	**FWHM [nm]**	**Overall quality factor** ***Q***	**In-cavity peak power [mW]**	**Peak SH efficiency [%/W]**
EC#1	0.0076	1.25 × 10^5^	13.63	218
EC#2	0.0068	1.38 × 10^5^	15.85	202.9
EC#3	0.006	1.50 × 10^5^	16	165.1

Therefore, Fig. 9(b) indicates that large values of the resonance mismatch $({\lambda }_{SH}-{\lambda }_{SH}^{0})$ can inhibit the SH generation. However, this drawback can be avoided by controlling appropriately the longitudinal resonance order for both pump and SH wave. In this sense, Table 4 summarizes the pump and SH resonance wavelengths for different values of the p-i-n distribution period, assuming $R$ = 249.97 µm.

**Table 4:** Spectral resonances for Pump and SH waves.

PIN distribution period $\Lambda$ [µm]	Resonance wavelength [µm]	
	Pump	SH wave
1.4396	2.2811	1.1409
1.4409	2.2841	1.1423
1.4422	2.2870	1.1436
1.4435	2.2900	1.1450
1.4449	2.2930	1.1464
1.4462	2.2959	1.1477
1.4475	2.2989	1.1491

Finally, we have theoretically demonstrated that the EFISHG effect in MRR can induce an efficiency that is from ~2 to 46 times larger than the value obtained in straight waveguides, when changing the ring radius from 699.98 to 199.95 µm, and holding the same fabrication and operative conditions, i.e. waveguide cross section, input pump power, reverse bias voltage, and nonlinear section length. Moreover, our simulations show that assuming $R$ = 249.97 µm, ${P}_{in}\,$ = 25 mW, EC#1, and ${L}_{NL}$ = 1 mm, an efficiency as large as 218%/W can be obtained. However, since the coupling interaction length has been estimated as 34 µm, we can increase the nonlinear section length up to ${L}_{NL}$ = 1.52 mm so as to achieve $\eta$ = 395%/W, corresponding to an absolute power conversion efficiency ${\eta }_{ab}$ ≈10%. It is worth noting that our values are comparable with the results proposed in ref. 33, where a maximum absolute power efficiency ranging between 12% and 26% has been obtained in AlN MRR with an input pump power of 27 mW.

### EFIM effect in microring resonator

The goal of this section is to investigate the EFIM process in the SOI technology platform. In particular, we propose the implementation of the EFIM process in the one-chip (Si) architecture sketched in Fig. 1, where the co-propagating pump and idler waves interact inside the microcavity to produce up/down spectral shifts. The EFIM device is obtained by realizing a periodic distribution of reversed biased p-i-n junctions, with a period *Λ* and 50% duty cycle, a QPM waveguide that induces the SFG (DFG) effect and generating the desired telecom (mid-IR) signal output.

Consequently, we report, as first, on the investigation of the architecture sketched in Fig. 1(a), choosing a pump wave at the wavelength *λ*_1_ ≅ 2.29 µm, and the mid-IR idler LD operating at *λ*_3_ ≅ 3 µm where Si does not suffer from any degenerate TPA effect. The telecom SFG signal is then generated at *λ*_2_ ≅ 1.298 µm.

Generally speaking, large values of *W* are required to well confine the guided modes inside the intrinsic layer, reducing the loss effects induced by the lateral doped-Si layers (see 1(b)), as well as inducing the mode propagation in a large spectral range from near-IR (1.298 µm) to mid-IR (3 µm). Conversely, low values of waveguide width are useful in order to induce a larger DC electric field (${E}_{DC}^{x}$) inside the waveguide with the application of low values of the bias voltage. As a trade-off among these conflicting requirements, we choose a reference waveguide with *W* = 1 µm, *H* = 0.5 µm and *t* = 100 nm.

Our investigations on the p-i-n diode reveal that the DC electric field inside the intrinsic Si layer assumes an average value ranging from 19.10 to 36.54 V/ µm, when changing the reverse bias voltage from ${V}_{bias}$ = −15 V to −30 V. Moreover, numerical p-i-n simulations performed under optical generation rate (similar to Figs 3 and 4) show that the TPA effects produce a carrier accumulation low enough to inhibit any detrimental screening effect on the electric field-induced second order susceptibility. In this context, assuming ${\chi }_{xxx}^{(3)}$ = 6 × 10^−19^ m^2^ V^−2^ at the pump wavelength of 2.29 µm^22, 23^, the electric field induced-${\chi }_{eff}^{(2)}$ changes from 32.95 to 63.02 pm/V, according to Eq. (11) (see the Method section).

**Figure 10:**
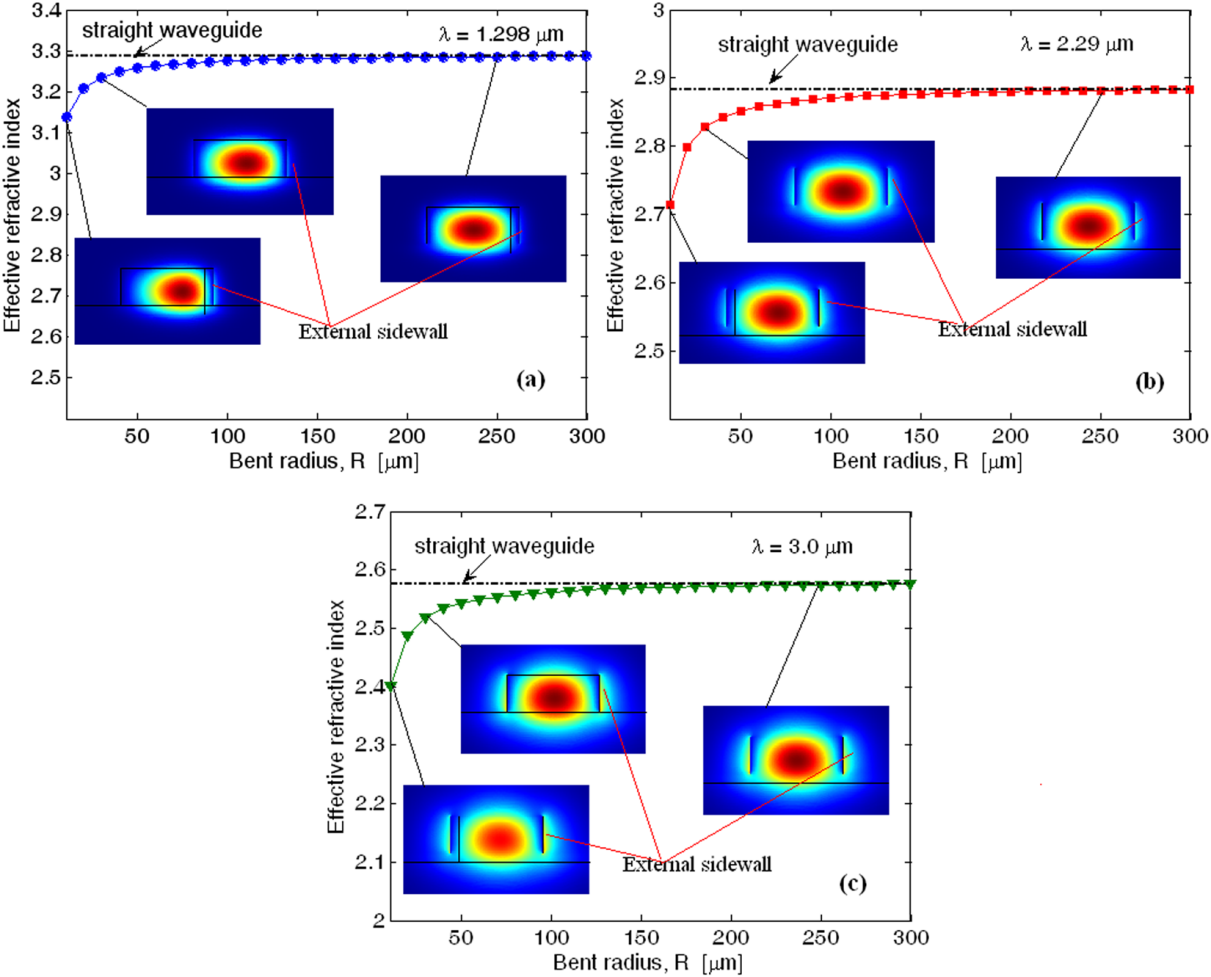
Effective refractive index as a function of the bent radius for the fundamental quasi-TE mode (**a**) at *λ* = 1.298 µm; (**b**) at *λ* = 2.29 µm; (**c**) at *λ* = 3 µm; The insets represent the space distribution of the dominant electric-field component for three different bent radius: *R* = 10, 30 and 250 µm.

According to the architecture sketched in Fig. 1(a), the SFG output power ($${P}_{out}^{SFG})$$ around 1.298 µm has been calculated, assuming the pump, the mid-IR idler, and the telecom SFG waves are aligned as TE_00_ modes in order to maximise the overlap integral given by Eq. (11). The effective refractive index for the fundamental quasi-TE modes as a function of the bend radius is shown in Fig. 10(a–c) at *λ* = 1.298, 2.29 and 3.0 µm, respectively. Horizontal lines indicate the effective refractive index values for straight waveguides with the same cross-section and operating at the same wavelengths. Moreover, each plot contains three insets representing the modal distribution of the dominant electric-field component for three different values of the ring radius: $$R$$ = 10, 30, and 250 µm.

The plots indicate that the bending influence is significant up to $R$ = 50 µm and can be considered negligible for ring radius larger than 80 µm where the effective refractive index starts to approach the value relevant to straight waveguides. Thus for $R$ < 50 µm the bending effect manifests itself as a shift of the optical mode distribution towards the external waveguide sidewall (see inset at $R$ = 10 µm), inducing an increasing of the overlap between optical distribution and lateral roughness. Moreover, low-order roughness harmonic can be involved in the phase matching condition, inducing modal-coupling losses at the mid-IR wavelength. However, ring resonators with $R$ > 200 µm (as in our device) suffer from modal-coupling losses negligible with respect to the carrier-induced loss ${\alpha }_{i}^{carrier}$. In addition, the scattering loss coefficient can be assumed as low as ${\alpha }_{i}^{scat}$ ≈2 dB/cm.

**Figure 11:**
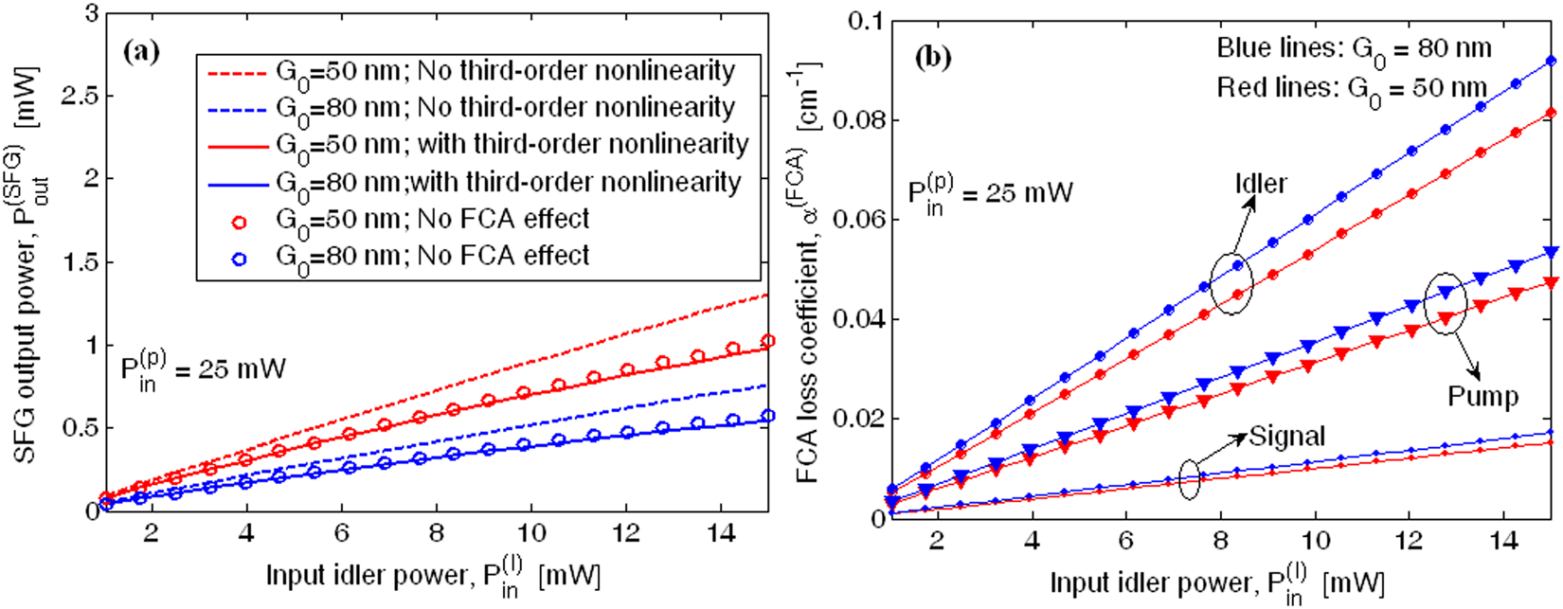
(**a**) Output SFG power versus input idler power for different values of the coupling gap; (**b**) FCA loss ciefficients versus input idler power for different values of the coupling gap.

The numerical results have been obtained by solving the equations system (1–4) where all nonlinear third order effects (TPA, SPM, XPM, FCA and plasma dispersion) are included. In this context, Fig. 11(a) shows the SFG output power as a function of the input idler power for different values of the coupling gap ${G}_{0}$, assuming ${P}_{in}^{(p)}$ = 25 mW,$\,{L}_{NL}$ = 1 mm, ${V}_{bias}$ = −20 V (${\chi }_{eff}^{(2)}$ = 43.08 pm/V), ${\tau }_{eff}$ = 0.5 ns (worst case),$\,{\alpha }_{1}$ = 4.03 cm^−1^, ${\alpha }_{2}$ = 0.68 cm^−1^, and ${\alpha }_{3}$ = 4.6 cm^−1^, respectively, where, following approach discussed in the previous section, we have theoretically estimated ${\alpha }_{p}^{carrier}$ = 3.53 cm^−1^, ${\alpha }_{s}^{carrier}$ = 0.18 cm^−1^, ${\alpha }_{I}^{carrier}$ = 4.1 cm^−1^, assuming ${\alpha }_{i}^{prop}$ ≈0.5 dB/cm.

These simulations have been performed in the optimal conditions, combining a perfectly fulfilled quasi-phase-matching condition (${\rm{\Delta }}{k}^{\text{'}}=0$, i.e. *Λ* = 2.421 µm, as in the Method section) with resonance matching for all waves involved in the parametric process. The plot includes three different operative conditions: i) the ideal condition (dashed lines), where the third order nonlinearities are neglected (only the SFG process occurs); ii) the condition where the third order effects (TPA, SPM, XPM) are all included, but without FCA (markers); iii) the condition where the third order effects are all included together with the FCA (solid line). It is worth outlining that, although the pump and idler waves do not suffer from any TPA effect (above the cut-off), the nonzero value of the degenerate and nondegenerate TPA coefficients at the telecom wavelengths induces detrimental effects that are not negligible. In particular, by estimating the values ${\beta }_{1,1}^{TPA}$ = 0,$\,{\beta }_{2,2}^{TPA}$ = 0.6 cm/GW, ${\beta }_{3,3}^{TPA}$ = 0, ${\beta }_{1,2}^{TPA}$ = 0.5 cm/GW, ${\beta }_{1,3}^{TPA}$ = 0, and ${\beta }_{2,3}^{TPA}$ = 0.25 cm/GW^23^, a lowering up to −2.33 dBm can be recorded in the SFG output power with respect to the ideal case. In addition, the plot indicates that the output SFG power is mainly limited by the TPA effects, producing the FCA effect with only a weak contribution for input idler power above 8 mW (see solid lines and markers). Furthermore, Fig. 11(b) shows that the FCA losses are always less that the linear loss coefficients, for the range of the input idler power considered.

However, it is not easy to simultaneously realise the resonance matching for all waves involved in the parametric process, but it is possible to design the cavity resonator to induce a distribution of the resonant angular frequencies such as the frequency difference between only two of the three waves contains an integer number of FSR values. Under this condition, a discrete set of resonator ring radius $R$ can be adopted according to Eq. (10):

10 $$R=|\frac{l\cdot c}{({\omega }_{i}^{0}\cdot {n}_{eff,i}-{\omega }_{j}^{0}\cdot {n}_{eff,j})}|\quad i,j=p,I,s;\,\,i\ne j$$

where *l* is the integer number indicating the difference between the longitudinal order for the *i*, and *j* waves, respectively. Therefore at the first stage, perfectly fulfilled resonance matching condition is obtained for *i*, and *j* waves, respectively. Then, the remaining optical wave involved in the parametric process, *k*, (*k* = *p*, *I*, *s*) will suffer from a resonance mismatch given by:

11 $${\rm{\Delta }}{\omega }_{k}=|{\omega }_{i}^{0}\pm {\omega }_{j}^{0}|-{\omega }_{k}^{0}$$

where ${\omega }_{i}^{0}$, ${\omega }_{j}^{0}$, and$\,{\omega }_{k}^{0}$ represent the cavity resonance frequencies, and the sign ± depends on the choice made for the *i* and *j* waves.

**Figure 12:**
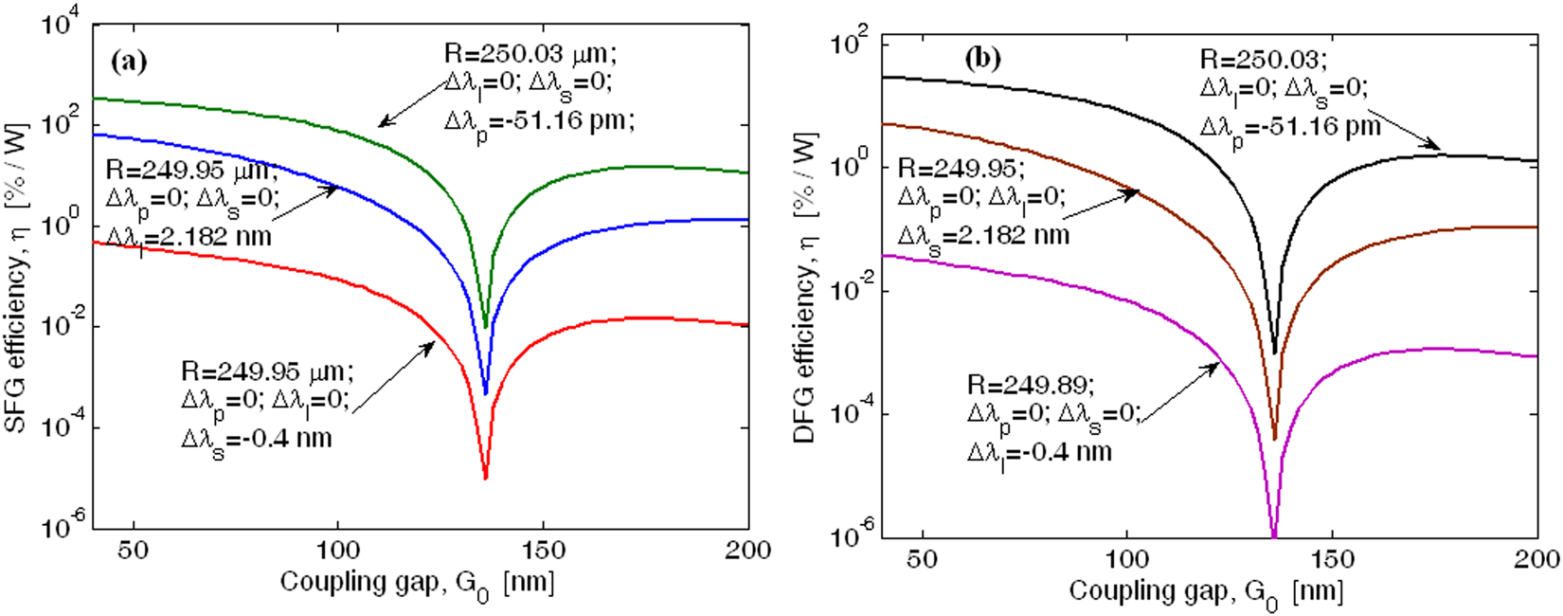
EFIM efficiency as a function of the coupling gap, for different resonance mismatch conditions. (**a**) SFG parametric process. (**b**) DFG parametric process.

Following Eqs (1–4), a parametric investigation of the SFG efficiency in microring resonators as a function of the coupling gap has been carried out. Numerical results are shown in Fig. 12(a), where the output SFG efficiency $\eta ={P}_{out}^{(SFG)}/({P}_{in}^{(p)}{P}_{in}^{(I)})$ is plotted as a function of the coupling gap ${G}_{0}$ for different resonance mismatch conditions, assuming ${P}_{in}^{(p)}$ = 25 mW, ${P}_{in}^{(I)}$ = 8 mW,$\,{L}_{NL}$ = 1 mm,$\,{\alpha }_{1}$ = 4.03 cm^−1^, ${\alpha }_{2}$ = 0.68 cm^−1^, and ${\alpha }_{3}$ = 4.6 cm^−1^. Moreover, a ring radius of 250 µm has been considered as the starting point for the design procedure. The plot reveals that the SFG process can be induced with a maximum efficiency of 454.4 %/W in the condition for which the idler and signal waves are perfectly matched to the cavity resonances ($R$ = 250.03 µm), inducing a consequent pump off-resonance of about −51.16 pm. Moreover, the worst case is recorded when the perfect resonance matching involves the pump and idler waves ($R$ = 249.95 µm), inducing for the telecom signal a mismatch of about −0.4 nm.

**Figure 13:**
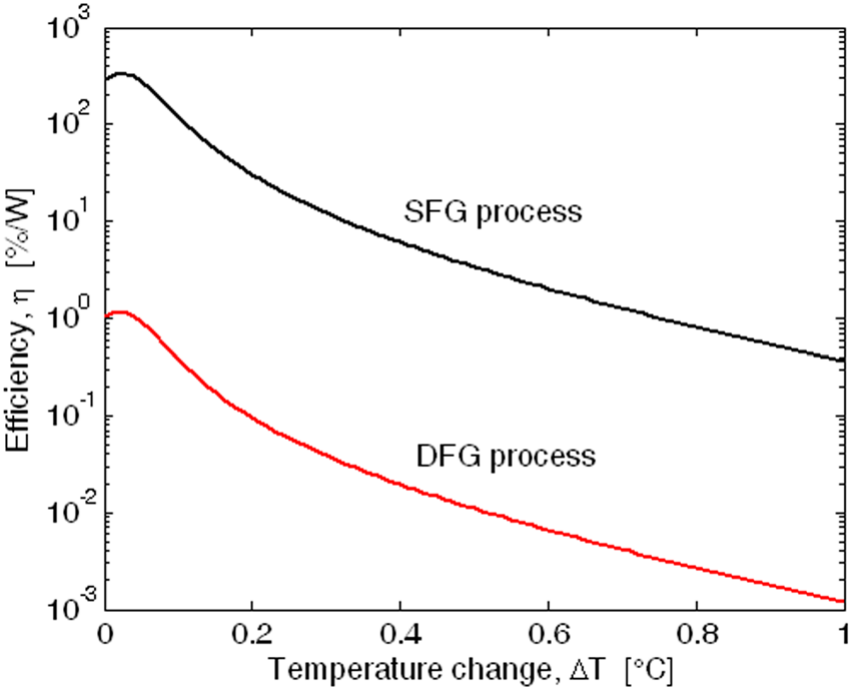
Efficiency *η* for SFG and DFG processes as a function of the temperature changes.

With the aim of analyzing the symmetry between the SFG and DFG processes, we have simulated both effects by setting the same geometrical and physical parameters for each process considered. In this sense, Fig. 12(b) shows the DFG output efficiency as a function of the coupling gap ${G}_{0}$ for different resonance mismatch conditions. Thus, ${\tau }_{eff}$ = 0.5 ns,$\,{L}_{NL}$ = 1 mm,$\,{\alpha }_{1}$ = 4.03 cm^−1^, ${\alpha }_{2}$ = 0.68 cm^−1^, and ${\alpha }_{3}$ = 4.6 cm^−1^, *Λ* = 2.421 µm, ${P}_{in}^{(p)}\,(@\,2.29\,\mu m)$ = 25 mW, and ${P}_{in}^{(I)}(@\,1.298\,\mu m)$ = 8 mW have been adopted in our simulations, in order to induce the DFG signal at 3$\,\mu m$. Therefore, the plot confirms that the DFG output efficiency can be maximized if the ring resonator is designed to match the idler and signal waves at the cavity resonance. Moreover, the results show that the DGF parametric process can be induced with an efficiency of 29.7 %/W, 11 times less than that of the SFG process. This asymmetry originates from a different influence of linear and nonlinear losses. In particular, the DFG effect generates a mid-IR signal at 3 $\mu m$ where the degenerate TPA effect is equal to zero but, at the same time, the idler wave at 1.298 µm suffers from a relatively high TPA-induced depletion effect. An opposite trend can be observed in the case of the SFG process. Consequently, the worst case is recorded for the parametric process inducing higher level of pump and idler depletion effect (DFG in our case). Finally, thermal effects on the SFG and DFG processes are shown in Fig. 13, where the efficiency $\eta$ is plotted as a function of the thermal changes (${\rm{\Delta }}T$) with respect to the room temperature (${T}_{0}$). Through the generalized Sellmeier equation^34^ and FEM simulations, we have estimated the coefficient $d{n}_{eff}/dT$ as 2.05 × 10^−4^ °C^−1^, 1.92 × 10^−4^ °C^−1^, and 1.79 × 10^−4^ °C^−1^ at *λ* = 1.298, 2.29 and 3.0 µm, respectively. Moreover, ${P}_{in}^{(p)}$ = 25 mW, ${P}_{in}^{(I)}$  = 8 mW,$\,{L}_{NL}$ = 1 mm, and a coupling gap ${G}_{0}$ = 50 nm has been considered in our simulations. The curves shows a maximum $\eta$ for ${\rm{\Delta }}T$ = 0.03 °C, where the positive pump resonance mismatch induced by thermal change partially compensates the initial pump off-resonance of about −51.16 pm, occurring in the device designed at room temperature. However, the plot shows clearly how thermal changes are significant, therefore low input power (as assumed) and temperature control are required.

## Conclusions

In this paper, a detailed modeling has been implemented for investigating the electric-field induced mixing effect, in which the up/down frequancy conversion processes are obtained inducing an effective second order susceptibility via the periodic spatial distribution of reverse biased p-i-n junctions. Moreover, the feasibility and performance of an integrated microring resonator based on the EFIM effect has been proposed and simulated in SOI waveguides. Thus, several operative conditions for the p-i-n diodes when changing the optical generation rate and the reverse bias voltage have been considered to estimate the influence of the TPA effect on the screening electric field induced by the carrier accumulation in the intrinsic waveguide region. Therefore for the SOI waveguides considered, the analysis has revealed that the TPA effect induces a reduction of the output efficiency but does not compromise the behaviour of the integrated EFIM MRR. Indeed the screening electric field induced by carrier accumulation is low enough to avoid important influences on electric field-induced second order susceptibility. Numerical simulations have revealed an EFISHG efficiency as large as 627.54 %/W when assuming $R$ = 249.97 µm, ${P}_{in}\,$ = 25 mW, *G*_0_ = 66 nm and ${L}_{NL}$ = 1.52 mm.

Finally, for the DFG/SFG conversion, from telecom idler (1.298 *μm*) to mid-IR (3 *μm*) and from Mid-IR(3 *μm*) to telecom signal (1.298 *μm*), an efficiency of 85.9%/W and 454.4 %/W has been obtained in the silicon microring resonator ($R$ = 249.97 µm), respectively. The techniques described here apply also to C-band telecom idlers/signals at 1.55 um by utilizing longer-wave mid-infrared pumps. Our physical model applies to all waveguides made of centro-symmetric semiconductors such as diamond, 3C-SiC, Si and Ge.

## Methods

Numerical estimations of ${\chi }_{eff}^{(2)}$ is needed to apply the equations system (1–3). For an applied DC and optical fields along the x direction, the nonlinear displacement current for the silicon reveals that the bulk induced electric field second order susceptibility can be expressed as ${\chi }^{(2)}=3{\chi }_{xxx}^{(3)}{E}_{DC}^{x}$, where ${\chi }_{xxx}^{(3)}$ is the third order susceptibility of the intrinsic silicon layer, and ${E}_{DC}^{x}$ represents the DC electric field applied along the *x*-direction^22^. In this context, the effective second order nonlinear susceptibility that influences the energy change between pump, idler, and signal waves can be determined by means of the overlap integral given in Eq. (11):

11 $${\chi }_{eff}^{(2)}=3{\chi }_{xxx}^{(3)}\frac{{\int }_{intrinsicregion}{({e}_{s}^{x})}^{\ast }{e}_{p}^{x}{e}_{I}^{x}{E}_{DC}^{x}dx}{{\int }_{allspace}{({e}_{s}^{x})}^{\ast }{e}_{p}^{x}{e}_{I}^{x}dx}$$

where ${e}_{i}^{x}$ ($i=1,2,3$) indicates the *x*-component of the electric field relevant to the *i* ^*th*^ optical guided mode involved in the process.

According to the architecture of Fig. 1(a), the nonlinear device is obtained using alternating reverse-biased and unbiased p-i-n junctions having a spatial period $\Lambda$ and duty cycle $D={L}_{b}/\Lambda$. As a result, the ${\chi }_{eff}^{(2)}$ parameter contains a periodic modulation $g(z)$ (where $z$ is the curvilinear coordinate along the ring resonator) with period $\Lambda$, and duty cycle $D$, where ${L}_{b}$ is the length within the period containing the nonzero second order nonlinear coefficient. By approximating this periodic modulation with the Fourier series, we obtain Eq. (12):

12 $$g(z)=\sum _{m=-\infty }^{\infty }{C}_{m}{e}^{jm\frac{2\pi z}{\Lambda }}$$

where the Fourier coefficients ${C}_{m}$ are given by Eq. (13):

13 $${C}_{m}=\frac{1}{\pi m}sin(\pi mD)$$

For a given process with the frequencies of the interacting waves varying around their central frequencies ${\omega }_{1}$, ${\omega }_{2}$, and ${\omega }_{3}$, it is possible to find a single reciprocal vector, ${K}_{m}=2m\pi /\Lambda$, which would maximize the parametric amplification process. In this sense, the nonlinear device should be designed to satisfy the quasi-phase matching condition, as implicitly adopted in Eqs (1–3). Thus, the condition ${\rm{\Delta }}k^{\prime} ={k}_{2}-{k}_{1}-{k}_{3}-2\pi /\Lambda$ = 0 has been assumed, where ${k}_{1}$, ${k}_{2}$, and ${k}_{3}$ are the wave-vectors of the three waves involved in the process.
